# Publisher Correction: Neurovascular coupling and oxygenation are decreased in hippocampus compared to neocortex because of microvascular differences

**DOI:** 10.1038/s41467-021-24833-y

**Published:** 2021-07-19

**Authors:** K. Shaw, L. Bell, K. Boyd, D. M. Grijseels, D. Clarke, O. Bonnar, H. S. Crombag, C. N. Hall

**Affiliations:** School of Psychology and Sussex Neuroscience, University of Sussex, Falmer, Brighton, United Kingdom

**Keywords:** Neuro-vascular interactions, Cardiovascular biology

Correction to: *Nature Communications* 10.1038/s41467-021-23508-y, published online 27 May 2021

The original version of this Article contained errors in Eq. (1), in which Hbt(t) was used as a numerator and denominator instead of Hbr(t).

Equation 1 incorrectly read:

$${\mathrm {CMRO}_{2}}(t)={\mathrm {CBF}}(t)\times \frac{{\mathrm Hbt}(t)}{{\mathrm Hbt}(t)}.$$

The correct form of Eq. (1) is:

1 $${{{\mathrm{CMRO}}}_{2}}(t)={\mathrm CBF}(t)\times \frac{{\mathrm {Hbr}}(t)}{{{{\mathrm{Hbt}}}}(t)}$$

This has been corrected in the PDF and HTML versions of the Article.

