# Supplementary material for: Neurovascular coupling and oxygenation are decreased in hippocampus compared to neocortex because of microvascular differences
Source: Nat Commun. 2021 May 27;12:3190. doi: 10.1038/s41467-021-23508-y (PMC8160329; doi:10.1038/s41467-021-23508-y)
Supplement: Supplementary file 1 — Supplementary Information [file 41467_2021_23508_MOESM1_ESM.pdf]

Supplementary Information: Neurovascular coupling and oxygenation are decreased in hippocampus compared to neocortex because of microvascular differences. Shaw et al.

Supplementary Figures

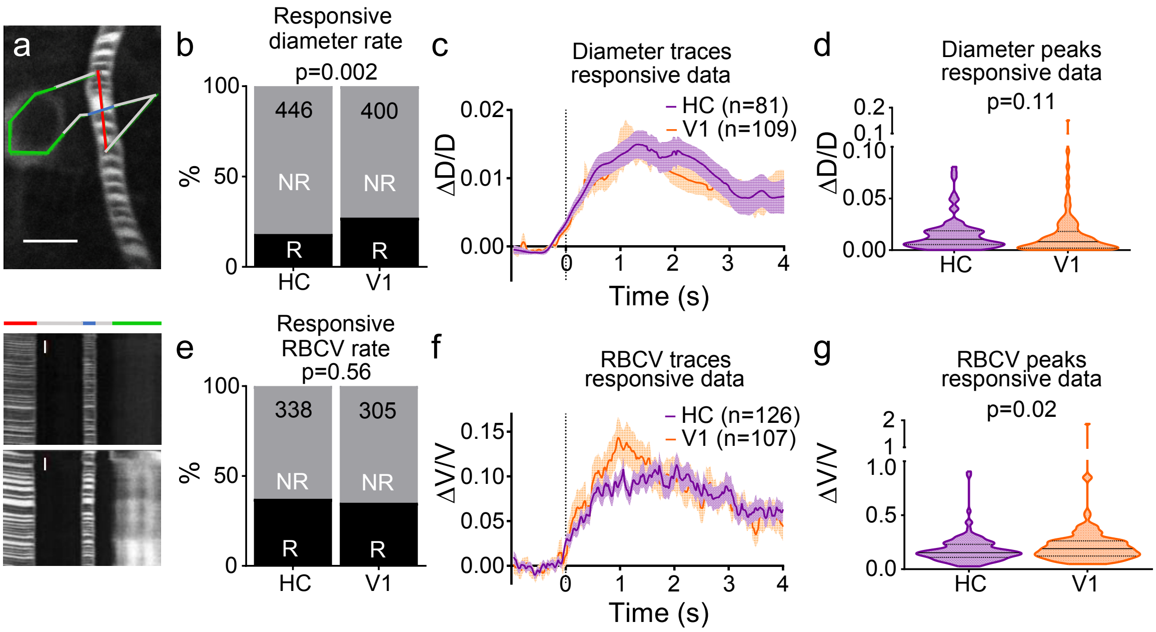

Supplementary Figure 1: RBC velocity increases less to local calcium increases in HC than V1.

(a) Upper panel: The line scan path (as inputted into the acquisition software – the actual trajectory will differ from that shown due to mirror inertia) was directed through the centre of a FITC dextran-filled vessel to extract RBC velocity (red) and a nearby GCaMP6f-positive cell (green) for calcium activations. Image is one representative example from 33 vessels scanned, and scale bar represents 10 µm. Lower panel: Line scans from trajectory indicated in upper panel, used to calculate RBC velocity (red line at top), capillary diameter (blue) and calcium changes (green) before (top) and during (bottom) calcium increase in the neuron. Scale bar represents 60 ms. **(b)** Capillaries were significantly more likely to dilate in V1 than HC (Chi-square test; NR: non-responsive, R: responsive). Numbers in bars refer to entire sample size. **(c)** Time course of dilation responses in HC and V1. **(d)** When they did occur, dilations were the same amplitude in V1 and HC (Mann-Whitney test, p=0.11). Data comes from 81 calcium events near 15 vessels from 5 animals in HC (range 3.3-5.9µm, average vessel diameter: 4.7µm) and 109 calcium events near 16 vessels from 5 animals in V1 (range 3.0-7.2µm, average vessel diameter: 5.1µm). **(e)** RBC velocity increased with equal frequency following a calcium event in HC and V1. Numbers in bars refer to entire sample size. **(f)** Time course of all significant increases in RBC velocity in V1 and HC **(g)** Increases in RBC velocity were larger in V1 than HC (Mann-Whitney test, p=0.02). Data comes from 126 calcium events near 15 vessels from 5 animals in HC (range 3.3-5.9µm, average vessel diameter: 4.7µm) and 107 calcium events near 16 vessels from 5 animals in V1 (range 3.0-7.2µm, average vessel diameter: 5.1µm). Vessels from the two regions were not different in size (t-test, p=0.32). Traces in c and f show mean +/- SEM, while all data are plotted in d and g. Source data are provided as a Source Data file. Outputs from statistical comparisons are provided in Statistics Report Tables SR5a-b.

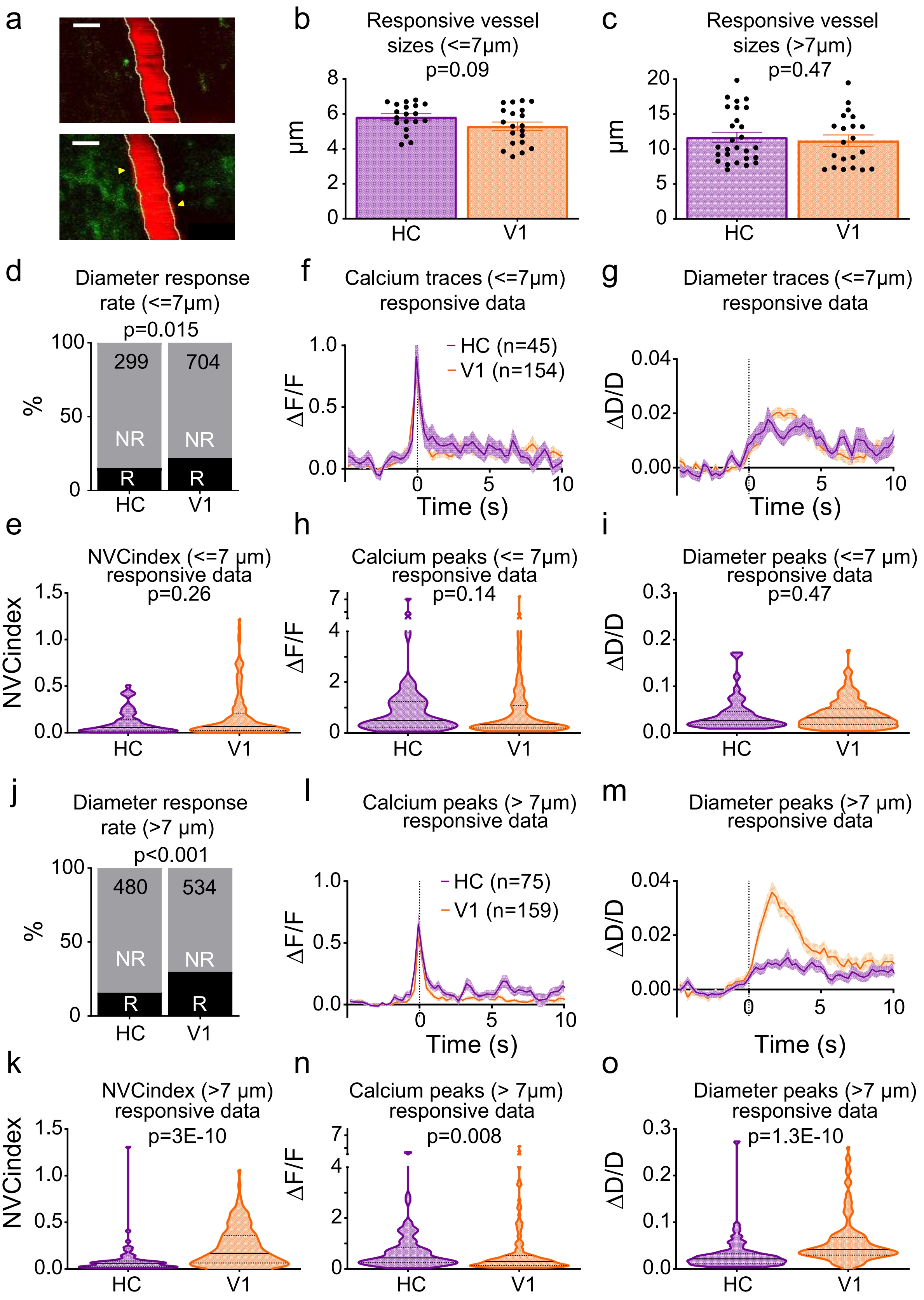

Supplementary Figure 2: Diameter responses are less frequent in smaller (<=7 µm) and larger (>7 µm) microvessels in HC, but responses are smaller in amplitude only in larger microvessels

(a) Texas Red dextran-filled vessel (red) and GCaMP6f-positive pyramidal neurons (green) from one recording in V1 before (top) and during (bottom) increases in neuronal calcium (to represent the 87 vessels with local neuronal calcium imaged). Yellow outlines show vessel before calcium event. Arrows indicate largest dilation. Scale bars represent 5 µm. The range of vessel diameters in **(b)** smaller (<= 7 µm) and **(c)** larger (>7 µm) vessels was not different between regions (unpaired t-tests comparing the individual vessels represented by the dots). **(d)** Vessels <=7 µm were more likely to dilate to preceding calcium events in V1

compared to HC (Chi-square test, numbers in bars refer to total sample size; NR: non-responsive, R: responsive), and both **(f)** calcium and **(g)** diameter traces were plotted for these responding vessels only. Of the responding vessels, there were no differences in **(e)** NVC_{index}, **(h)** calcium peaks or **(i)** diameter peaks between regions when individual calcium/diameter events were compared (HC N=45, V1 N=154). **(j)** Vessels >7 µm were more likely to dilate to preceding calcium events in V1 compared to HC (Chi-square test, numbers in bars refer to total sample size), and both **(l)** calcium and **(m)** diameter traces were plotted for these responding vessels only. Of the responding vessel dilations compared (HC N=75, V1 N=159), **(k)** NVC_{index} was larger in V1, due to **(n)** calcium peaks being smaller, and **(o)** diameter peaks larger in this region. Shaded error bands represented mean +/- SEM. P-values on plots are from Mann-Whitney U tests unless otherwise stated, and outputs from statistical comparisons can be found in Statistics Report Tables SR6a-b. Source data are provided as a Source Data file.

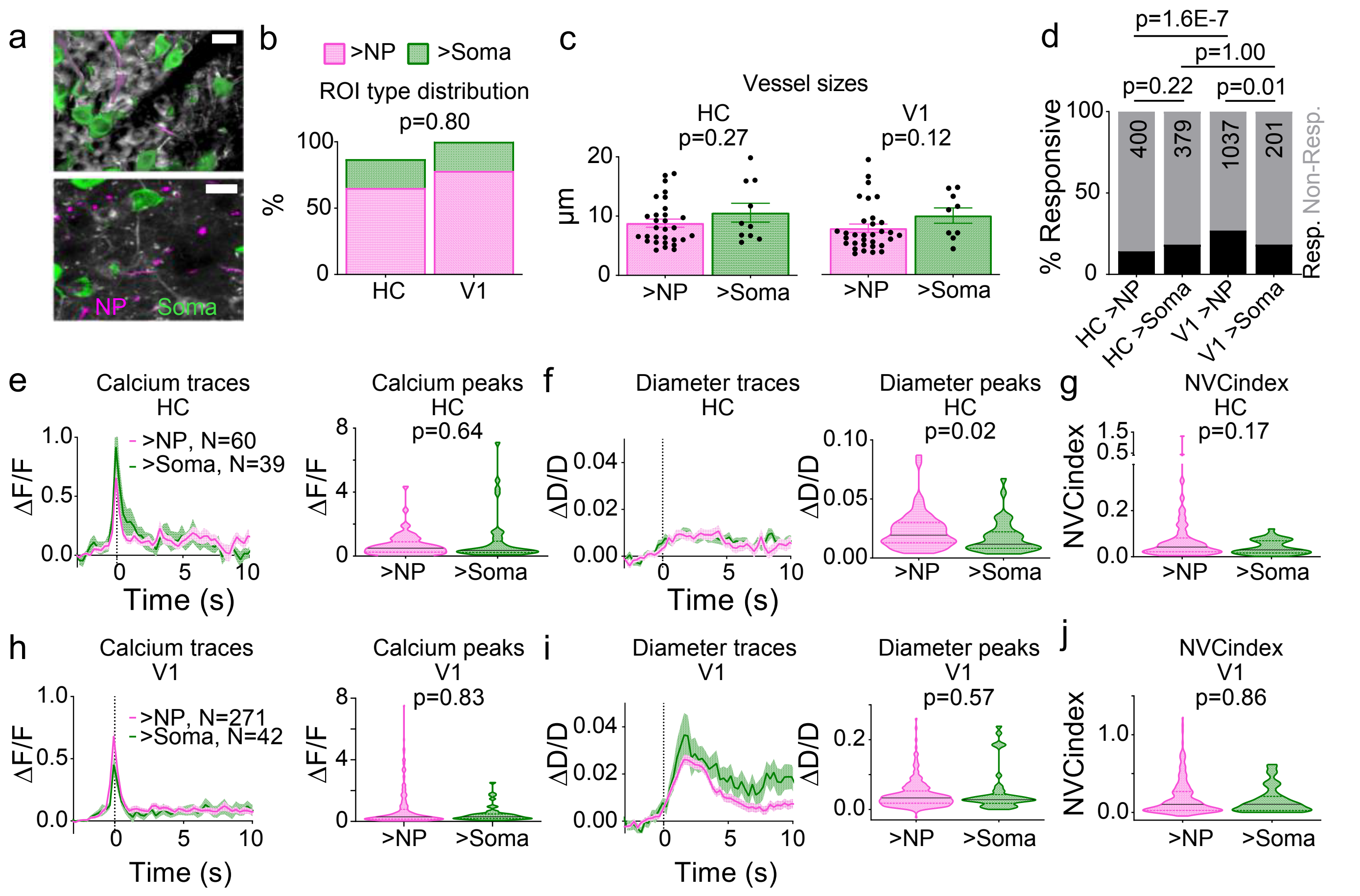

Supplementary Figure 3: The contribution of cellular input to vascular responses

(a) GCaMP6f-positive pyramidal neurons (white) with regions of interest (ROIs) categorised as soma (green) or neuropil (NP, pink) based on their morphology, for HC (top) and V1 (bottom). Images are one representative example from 41 local neuronal calcium recordings in HC and 46 in V1, scale bars represent 10 µm. **(b)** Neuronal calcium activity from individual recordings was classified by whether ROIs were majority NP or majority soma (>60% of the total ROIs; V1, N=313 calcium events, 7 mice; HC, N=105 calcium events, 6 mice; 15 recordings were excluded as there was no majority of either NP or soma). P value is from a 2x2 Chi-square contingency test. **(c)** Individual vessels were compared (HC N: >60%NP = 30, >60%Soma = 10; V1 N >60%NP = 32, >60%Soma = 9), and the sizes recorded in HC and V1 were the same for NP and soma recordings. **(d)** Response frequencies compared across brain regions, split by ROI type using the Cochran-Mantel-Haenszel 3D variant of a Chi-square test. Vessels near to NP were significantly more likely to dilate compared to those near somas in V1. Numbers in bars refer to total sample size per category. Average traces and peak responses of **(e)** calcium, and **(f)** corresponding vessels in HC for NP or soma. **(g)** NVC_{index} in HC did not differ between neuronal ROI types. Average traces and peak responses for **(h)** calcium and **(i)** corresponding vessels in V1 for NP or soma. **(j)** NVC_{index} did not differ in V1 by neuronal ROI type. Error bars and shaded error bands represent mean +/- SEM. Regional comparisons were made between individual calcium/dilation events in e-j (N specified on calcium traces in e & h). P-values are from Mann-Whitney U tests, unless otherwise stated (see Statistics Report Table 7a-c). Multifactorial ANOVAs also revealed that the regional differences in diameter responses and NVC indices were not affected by ROI type (Statistics Report Table 7d). Source data are provided as a Source Data file.

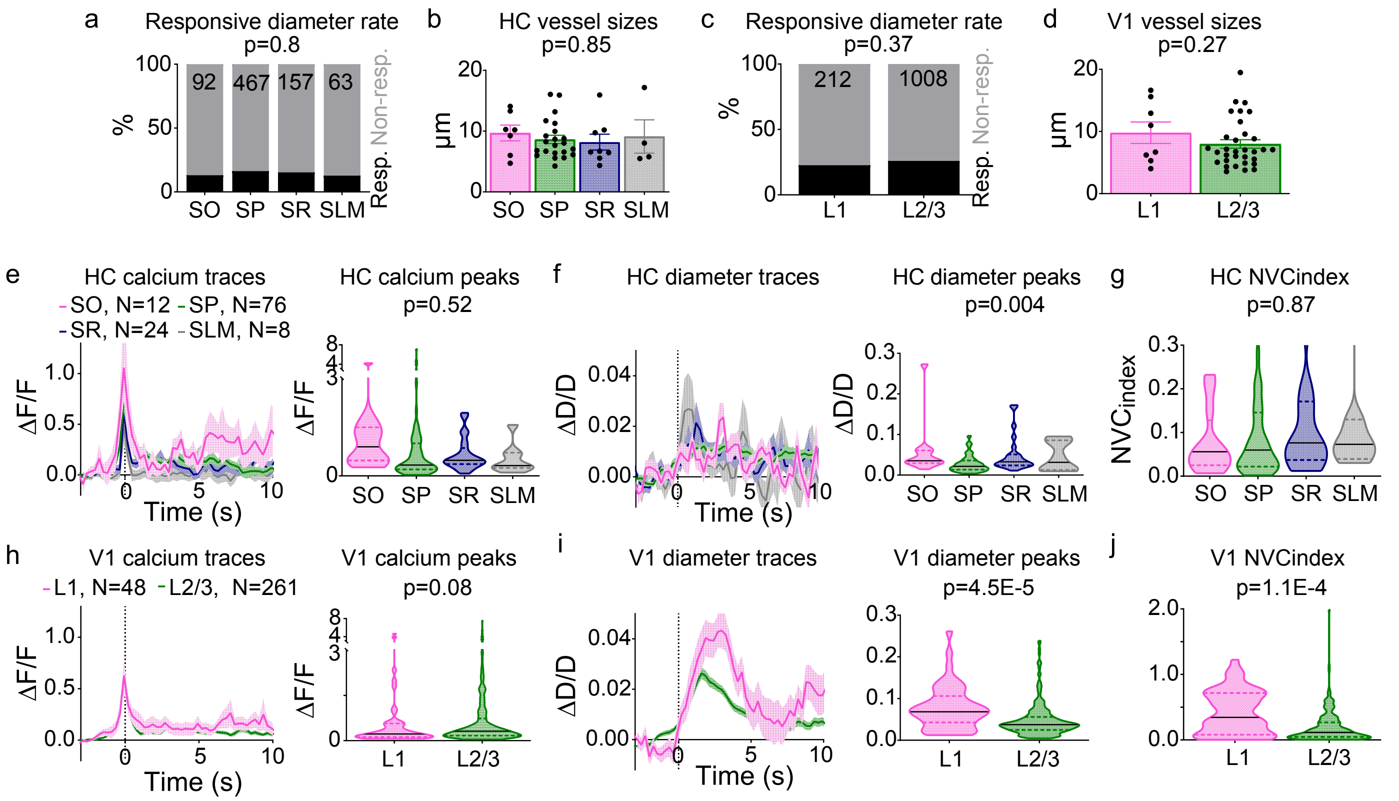

Supplementary Figure 4: The contribution of laminar organisation to vascular responses

Vessel recordings were separated by layer for HC (N=120 calcium events, 6 mice; SO = stratum oriens, SP = stratum pyramidale, SR = stratum radiatum, SLM = stratum lacunosum-moleculare) and V1 (N=309 calcium events, data from L4 was excluded due to low sample sizes (4 responsive events), 7 mice). Response frequencies were compared across layers for **(a)** HC (using the Cochran-Mantel-Haenszel 3D variant of a Chi-square test), and **(c)** V1 (using a 2x2 Chi-Square test). The numbers in the bars refer to the total sample size per condition. The vessel sizes sampled for each layer of **(b)** HC (SO N=7. SP N=22, SR N=8, SLM N=4) and **(d)** V1 (L1 N=8, L2/3 N=32) were not different between layers (one way ANOVA or independent sample t-tests comparing individual vessels). The average traces and their maximum peaks for **(e)** calcium, and **(f)** corresponding vessels in HC split by layer. Calcium peaks were not different between layers, and whilst a significant effect of layer was observed in HC (one-way ANOVA with Welch's correction, p=0.04), these could not be attributed to any specific layer comparisons (Games-Howell post-hoc, ns). **(g)** The NVC$_{index}$ did not differ by layer in HC. The average traces and their maximum peaks for **(h)** calcium and **(i)** corresponding vessels in V1, when split by layer. Vessel dilations were largest in L1. **(j)** The NVC$_{index}$ was largest in L1, due to bigger vessel dilations. NVC$_{index}$ in both L1 and L2/3 were nevertheless larger than in HC (one-way ANOVA with Welch's statistic and Games-Howell post-hoc comparisons: L1, L2/3 and average across HC; Statistics report 7i). Error bars and shaded error bands represent mean +/- SEM. Unless otherwise stated, p-values represent the result of independent sample Mann-Whitney U tests in V1, and one-way ANOVAs with Bonferroni or Games-Howell post-hoc comparisons in HC (see Statistics Report Tables SR8a-i). Source data are provided as a Source Data file.

Discussion of Supplementary Figs 3 and 4:

The subcellular elements of neurons are differently represented in the different layers, with a higher density of soma in SP of HC and L2/3 of V1 compared to the other layers. Our results across Supplementary Figs 3 and 4 show some indication that dendritic areas might show stronger neurovascular coupling: In HC, regions with more soma show smaller dilations (Fig S3f), and the soma-dense pyramidal layer also shows the smallest dilations (Fig. S4f). Furthermore, in V1, L1 showed larger diameter changes and NVC$_{indices}$ than L2/3 (Fig S4h-j). However, the evidence for stronger NVC in neuropil-dominated regions is equivocal. When the different sizes of local calcium responses are accounted for, by using the NVC$_{index}$, there are no longer any differences in HC between layers (Fig S4g), or between neuropil- and soma-dominated regions (Fig S3g). Additionally, in V1, the significant laminar changes between L1 and L2/3 (Fig S4h-j) cannot be attributed to different levels of soma vs neuropil, as when we explicitly tested this, there were no significant differences between soma or neuropil-dominated regions (Fig S3h-j). Therefore, the presence or absence

of laminar differences do not seem to be simply due to preferential vascular responding to signals from neuropil over soma.

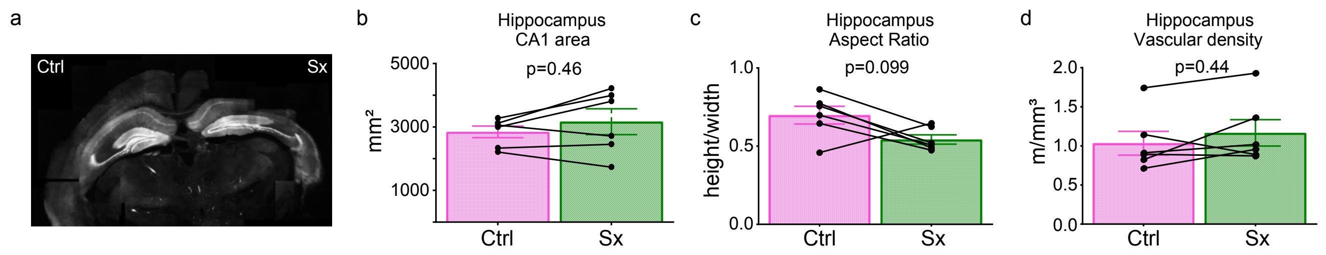

Supplementary Figure 5: Comparing the hippocampal surgical and control hemispheres

(a) Example 2D image of one brain slice showing CA1 on the control (i.e. non-surgical, left) and surgical (right) hemispheres (6 images taken from 6 slices/mice in total). Neither the area **(b)** nor the aspect ratio (height/width) **(c)** of CA1 significantly differed between the control and surgical hemispheres (paired ratio t-test comparing individual slices/animals between hemispheres, dots represent data from 6 slices/animals, and lines connect hemispheres from the same slice). **(d)** The vascular density also did not differ between control and surgical hemispheres (Wilcoxon matched-pairs test comparing individual slices between hemispheres, dots represent 6 slices from 3 animals). Error bars represent mean +/- SEM, and statistical outputs are presented in Statistics Report Table SR9. Source data are provided as a Source Data file.

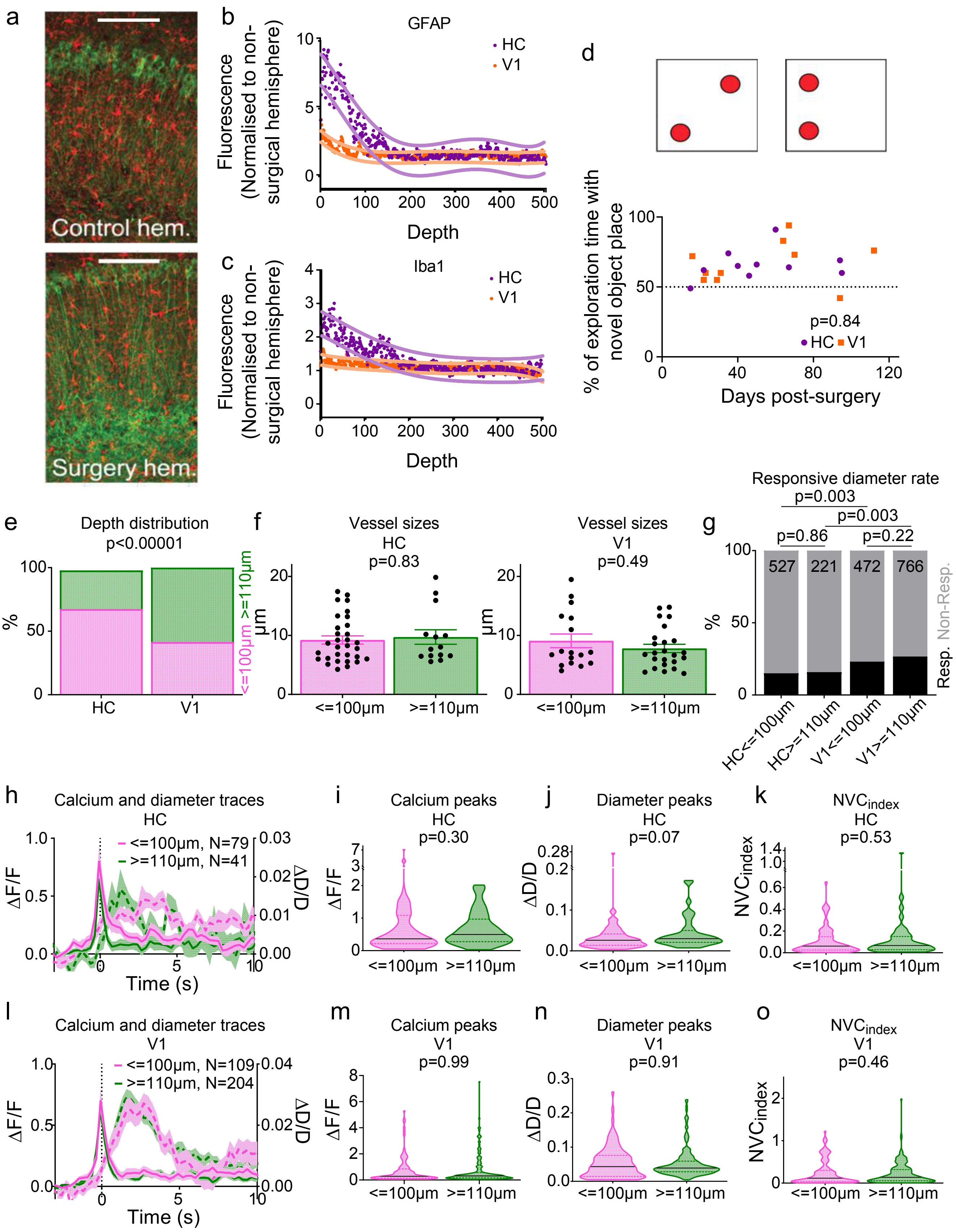

Supplementary Figure 6: The impact of hippocampal cranial window surgery compared to V1 cranial window surgery

(a) Example images from one mouse (of 6 total mice imaged) showing GCaMP6f-labelled neurons (green) and Iba1-labelled microglia (red) in the surgical and non-surgical hemispheres. Scale bar represents 200 µm. Data above and below 100 µm from the window was compared. Average profiles for intensity of **(b)** GFAP (left: HC, N=2 mice; V1, N=3 mice) and **(c)** Iba1 labelling (right: HC, N=3 mice, V1, N=3 mice). Data from each mouse is an average of inflammation profiles from 3-5 slices. Exponential fits to the data with 95% upper and lower confidence intervals are plotted. Inflammation levels were considered not to be

different at the point where confidence intervals overlapped (Iba1: 105 µm, GFAP: 110 µm). **(d)** Memory was assessed on a hippocampal-dependent novel object location task for mice which had undergone HC or V1 surgery (independent samples t-test, 10 animals per region). **(e)** Number and **(f)** sizes of vessels at different depths (N are taken from individual vessel diameters separated by depth/region, HC N: <100µm = 31, >110µm =14; V1 N: <100µm = 17, >110µm = 24). **(g)** Response frequencies were not different across imaging depth but were different between brain regions (Cochran-Mantel-Haenszel 3D variant of a Chi-square test, numbers in bars represent total number of calcium-aligned diameter events per group). The average traces for calcium (solid lines) and vessel diameter (dotted lines) **(h**: HC**; l**: V1**).** The corresponding maximum peak values for calcium and diameter traces **(i**: HC calcium**, j**: HC diameter; **m**: V1 calcium, **n**: V1 diameter**)**, and the NVC_{index} **(k**: HC**, o**: V1**)**, showed no differences based on imaging depth. HC: N=120 calcium events, 6 mice; V1: N=313 calcium events, 7 mice. Error bars and shaded error bands represent mean +/- SEM. P-values represent the result of independent sample Mann-Whitney U tests, unless stated, and are presented in Statistics Report Tables SR10a-c. Source data are provided as a Source Data file.

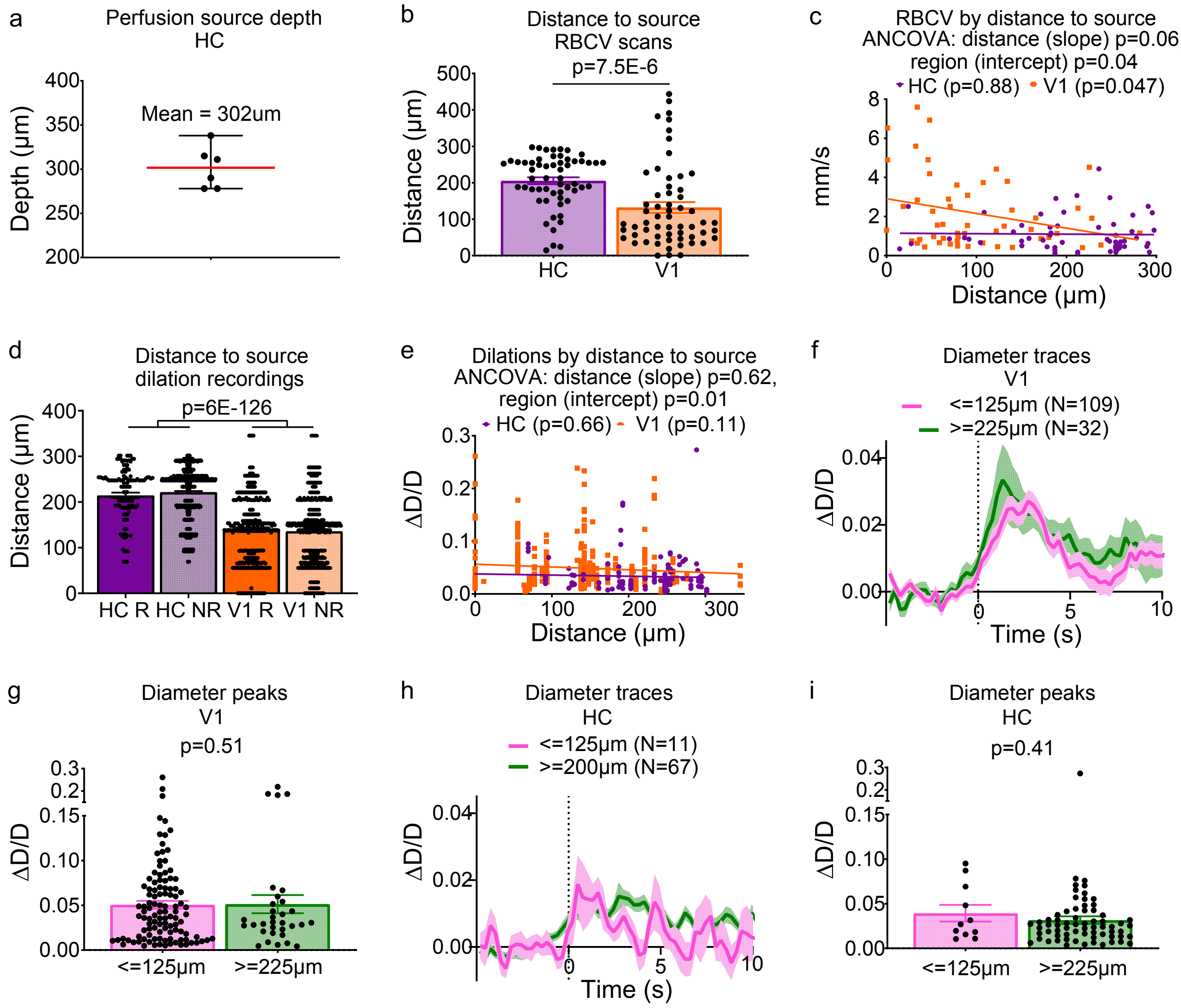

Supplementary Figure 7: The contribution of distance from the perfusion source on vascular responses

(a) The average depth below layer stratum oriens in CA1 hippocampus where the perfusion sources (feeding arteries) were located (N=6 *in vivo* Z-stacks from 6 animals). The average depth of these vessels (302 µm, red line) was used in subsequent plots to calculate the likely distance of recorded vessels in HC from their source. The vascular perfusion sources in V1 were the pial arteries. Error bar represents data range. **(b)** Line scan recordings of resting RBCV in HC (purple, N=57 vessels) and V1 (orange, N=59 vessels) were sorted by their distance from the vascular perfusion source. Vessels recorded from V1 were significantly closer to their perfusion source than those in HC (Mann-Whitney test). **(c)** The average resting RBCV plotted by their likely distance from the perfusion source showed a significant trend in V1 (p=0.047) but not HC (p=0.88, linear regression). In V1, vessels closer to the source had higher resting RBC velocity than those further away, contributing to the average faster velocity seen in this region. **(d)** Considering dilations, the distance to perfusion source of calcium event-responsive and non-responsive vessels in HC (left, purple) and V1 (right, orange). A multifactorial ANOVA revealed a significant regional difference in the distance between recorded vessels and their perfusion source (p=1.9E-74), but no differences between responsive dilation categories (R = responsive, NR = non-responsive, p=0.58), and no interaction (p=0.27). **(e)** There was no relationship between the size of vessel dilations and their distance from the perfusion source in HC (p=0.98) or V1 (p=0.11, linear regression). Diameter traces for vessels at distances closer to (<=125 µm) or further from (>=250 µm) the perfusion source in **(f)** V1 and **(h)** HC. Peak dilations of these vessels were not different near and far from the perfusion source in either **(g)** V1 (<=125µm N=32, >=225µm N=109) or **(i)** HC (<=125µm N=11, >=225µm N=67) (Mann-Whitney tests compared individual dilation peaks represented by dots). Unless stated, error bars and shaded error bands represent mean +/- SEM. Outputs from statistical comparisons are presented in Statistics Report Tables 11a-b. Source data are provided as a Source Data file.

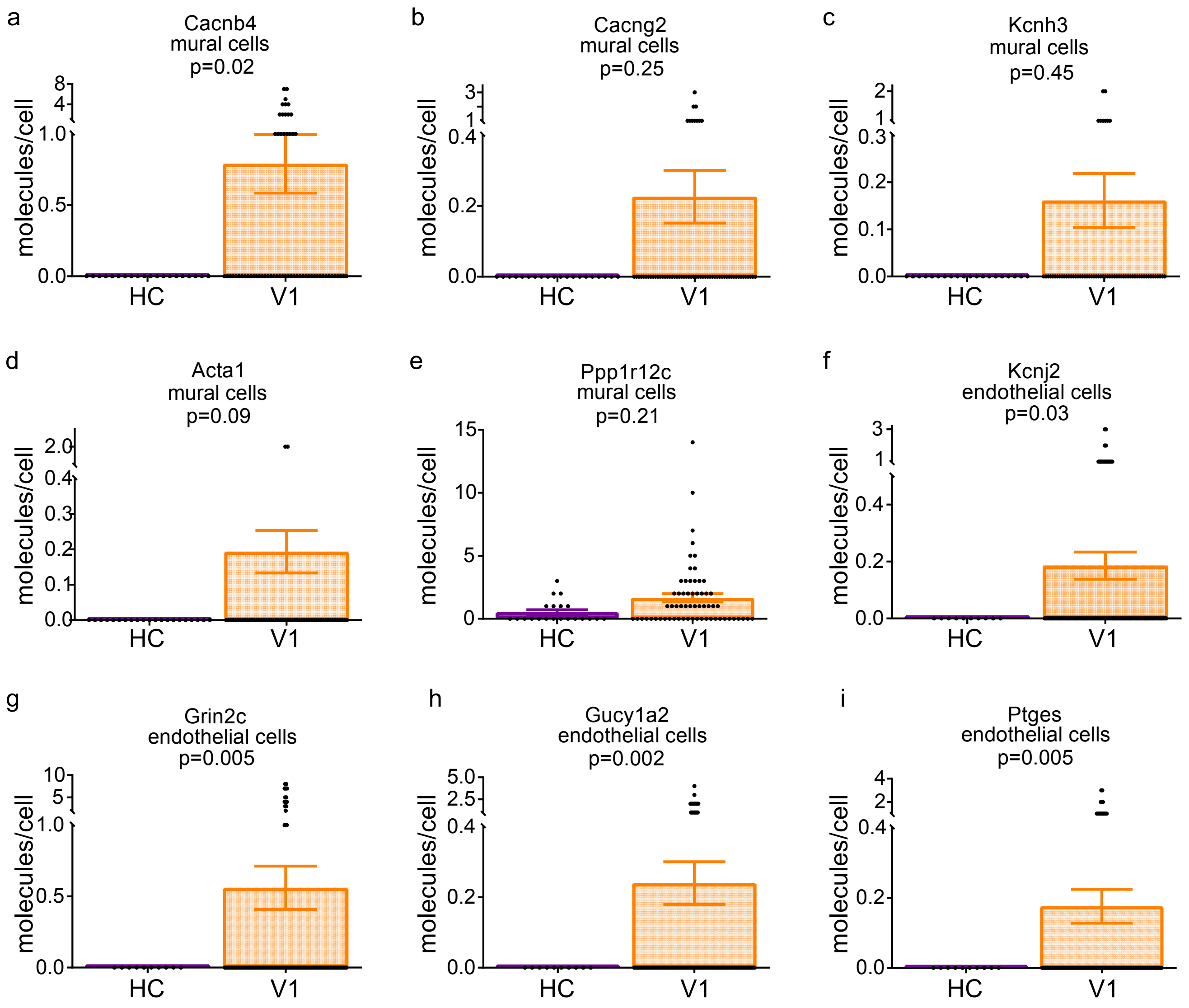

Supplementary Figure 8: Neurovascular pathways in vascular cells

The mRNA expression profile of mural and endothelial cells was tested across three broad categories known to link neurovascular function: ion channels, contractile machinery and neurovascular signalling pathways. A selection of the transcripts showing regionally-specific expression with (a,f-i) or without (b-e) correction for multiple comparisons. **(a)** Cacnb4 – beta 4 subunit of voltage-gated calcium channels, **(b)** *Cacng2* **–** stargazing calcium channel gamma subunit, **(c)** *Kcnh3 – voltage-gated potassium channel subunit*, **(d)** *Acta1 – skeletal muscle actin*, and **(e)** *Ppp1r12c – myosin phosphatase subunit* **(f)** Kcnj2 – inward-rectifying potassium channel Kir2.1, **(g)** Grin2c – NMDA receptor 2c subunit, **(h)** Gucy1a2 – soluble guanylyl cyclase alpha 2 subunit, and **(i)** Ptges – prostaglandin E synthase. Dots represent individual cells, which are compared between regions for all statistical comparisons. Error bars represent mean +/- SEM. P-values represent the result of independent sample t-tests with Holm-Bonferroni corrections for the multiple comparisons, original and adjusted p-values are presented in each of Supplementary Data Tables 4-5 which contain comparisons of all transcripts tested. Source data are provided as a Source Data file.

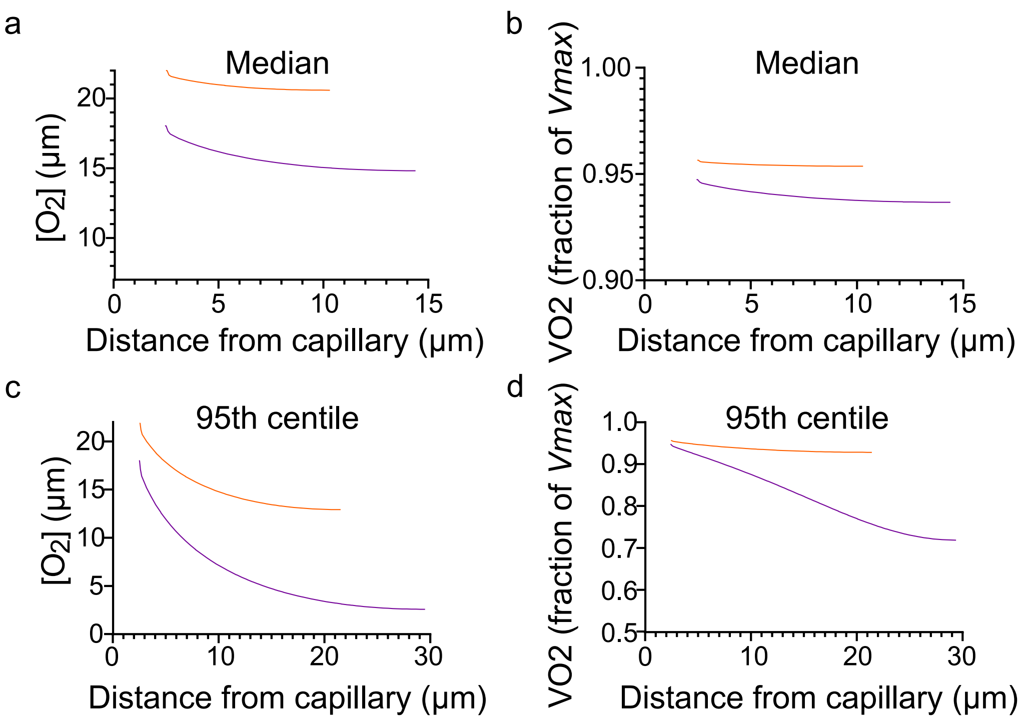

Supplementary Figure 9: Capillary pO2 estimated between two RBCs

Simulated time courses when capillary pO2 was estimated between two RBCs from initial conditions of zero [O_2] for tissue at **(a)** the median or **(c)** 95th centile distance from a capillary. O2 consumption rate as a fraction of Vmax (VO2) for **(b)** median and **(d)** 95th centile capillary spacing conditions, calculated from the oxygen profiles shown in (a) & (c). Lines represent a V_{max} of 2 for HC (purple) and V1 (orange).

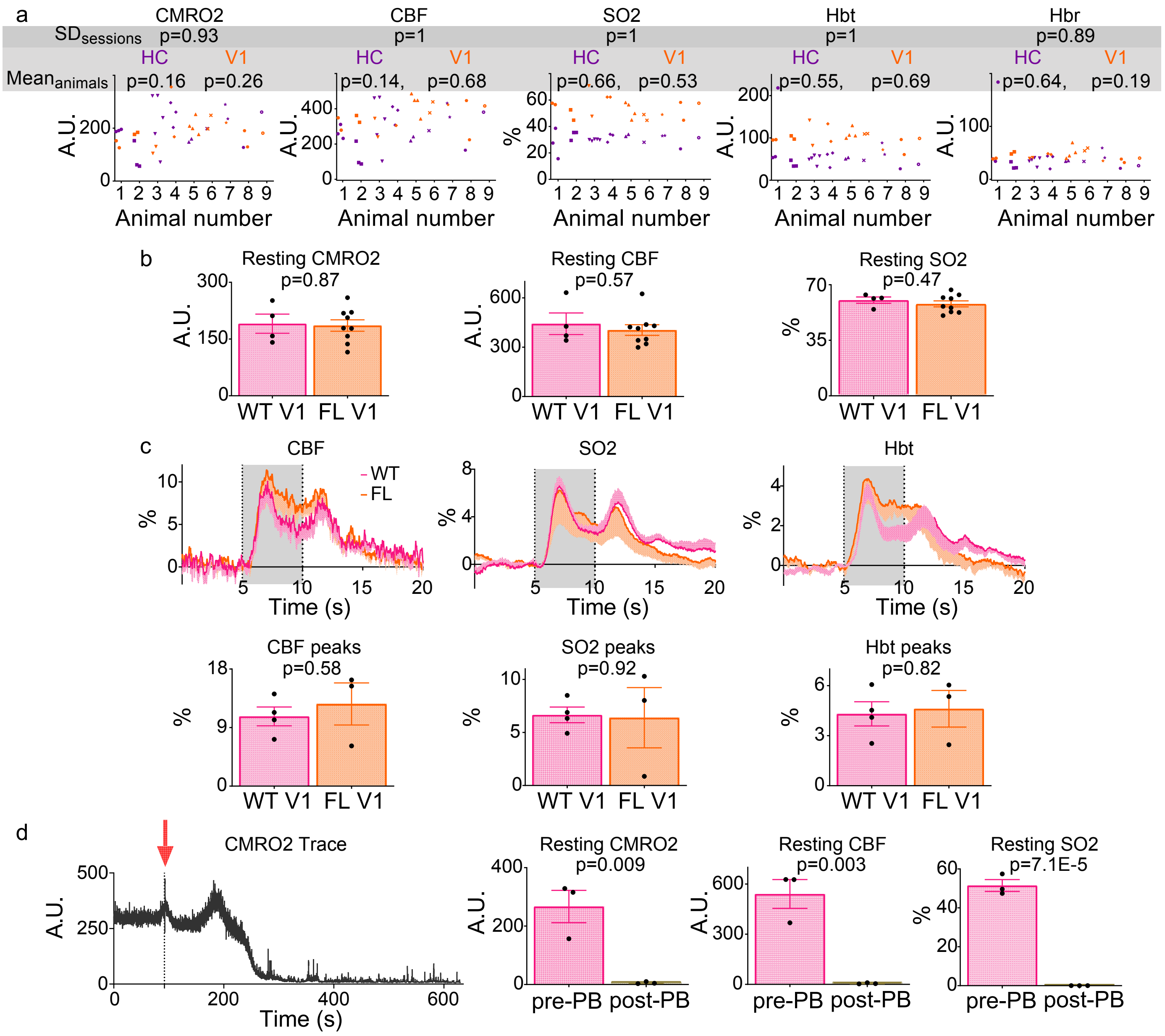

Supplementary Figure 10: Testing the reliability of oxy-CBF probe measurements
(a) The haemodynamic parameters measured by the oxy-CBF probe for $CMRO_2$, flux, SO_2, Hbt, and Hbr per recording session included in Figures 2a-c separated by animal (note that the regions included different animals). P-values below the graphs represent the results of one-way ANOVA tests for effects of animal ID on haemodynamics, and p-values below the graph titles represent the values of t-tests comparing the regional standard deviation between recording sessions after Bonferroni correction (for all animals with multiple sessions; see Statistics Report Table SR12a). **(b)** Resting $CMRO_2$, blood flow and SO_2 levels were compared for GCaMP6f-positive V1 mice (N=9 mice, taken from Figures 2a-c) to wild type V1 mice (non-fluorescent, N=4 mice across 12 sessions) to check for any effects of brain fluorescence on oxy-CBF measures. No significant differences were observed. **(c)** Stimulus-induced haemodynamic responses were also compared between wild type and GCaMP6f-positive V1 mice. Traces are taken from haemodynamic data averaged across animals (N=4 wild-type, N=3 fluorescent; error bars are SEM); and the peak responses of these traces per animal are represented by the dots in the bar charts below. **(d)** Wild-type mice were injected with pentobarbital (i.p.) while recording with the oxy-CBF probe to determine the baseline for the various parameters when blood flow ceased (N=3 mice). The black trace on the left of the bar plots shows an example continuous recording of $CMRO_2$, with the red arrow and dotted line representing when the injection was administered. Resting levels of $CMRO_2$, CBF and SO_2 were averaged during rest periods before the injection (i.e. alive), and for the last one minute of the recording for comparison (i.e. after death). Error bars and shaded error bands represent mean +/- SEM. Unless stated, p-value above graphs are taken from two-tailed unpaired t-tests (outputs presented in Statistics Report Table SR12b). Source data are provided as a Source Data file.

Supplementary Tables

Supplementary Tables 1-5: Single Cell RNA-Seq analyses

Data were taken from Zeisel et al [1] to allow comparison of mRNA transcript expression in neural, astrocytic and vascular cells of cortex and CA1 of hippocampus. The number of transcripts per cell were compared using Welch's t-tests, adjusted for multiple comparisons using a method equivalent to the Holm-Bonferroni method (for N comparisons, the most significant p-value is multiplied by N, the 2nd most significant by N-1, the 3rd most significant by N-2, etc.; corrected p-values are deemed significant if they are less than 0.05). Numbers of each cell type were as follows:

Pyramidal cells. Cortex: 398; HC: 941
Interneurons. Cortex: 169; HC: 126
Astrocytes. Cortex: 143; HC: 80
Mural cells. Cortex: 63; HC: 20
Endothelial cells. Cortex: 126; HC: 10

For neurons and astrocytes, mRNA transcripts were studied that code for neurovascular signalling molecules. For mural cells (smooth muscle cells and pericytes) we probed for contractile machinery, ion channels expected to modulate dilation (K^+ and Ca^{2+} channels) and receptors or synthetic enzymes for neurovascular signalling molecules.

**Supplementary Tables Key: (SD = standard deviation)**
Significant after correction for multiple comparisons
Significant before correction for multiple comparisons
Non-significant

Supplementary Table 1: Single cell mRNA comparisons for pyramidal cells

See Figures 4a-b

Gene name	Cortex Mean	Cortex SD	HC Mean	HC SD	p-value	Adj. p-value	Effect Size
Nos1	0.005	0.071	0.053	0.238	0.000	<0.001	0.237
Ptges3	2.422	2.842	3.077	2.858	0.000	0.001	0.229
Ptgs1	0.118	0.539	0.045	0.245	0.010	0.086	0.205
Pla2g4a	0.058	0.282	0.021	0.178	0.017	0.139	0.170
Ptgs2	0.261	0.763	0.163	0.577	0.021	0.149	0.154
Ptges	0.048	0.326	0.019	0.159	0.096	0.575	0.129
Ptges3l	0.168	0.545	0.121	0.364	0.115	0.577	0.110
Pld2	0.050	0.251	0.071	0.285	0.178	0.711	0.077
Ptges2	0.405	0.677	0.439	0.732	0.410	1.000	0.048
Pla2r1	0.013	0.132	0.010	0.097	0.685	1.000	0.027
Pld1	0.035	0.198	0.026	0.171	0.398	1.000	0.054

Supplementary Table 2: Single cell mRNA comparisons for interneurons

Gene name	Cortex Mean	Cortex SD	HC Mean	HC SD	p-value	Adj. p-value	Effect Size
Ptges	0.054	0.274	0.008	0.089	0.044	0.488	0.212
Ptges2	0.411	0.686	0.595	0.887	0.054	0.536	0.237
Nos1	0.065	0.331	0.040	0.196	0.405	1.000	0.092
Ptges3	3.911	4.044	3.794	3.693	0.796	1.000	0.030
Ptges3l	0.054	0.367	0.032	0.176	0.501	1.000	0.073
Pla2r1	0.006	0.077	0.000	0.000	0.319	1.000	0.102
Pld1	0.030	0.203	0.008	0.089	0.214	1.000	0.133
Pld2	0.155	0.424	0.111	0.316	0.312	1.000	0.115
Ptgs1	0.220	0.687	0.341	0.647	0.123	1.000	0.181
Ptgs2	0.012	0.109	0.000	0.000	0.158	1.000	0.145
Pla2g4a	0.030	0.230	0.032	0.217	0.940	1.000	0.009

Supplementary Table 3: Single cell mRNA comparisons for astrocytes

See Figure 4c

Gene name	Cortex Mean	Cortex SD	HC Mean	HC SD	p-value	Adj. p-value	Effect Size
Ptges3	0.483	0.786	1.513	2.882	0.002	0.040	0.561
Ptgs1	0.259	0.590	0.100	0.341	0.012	0.186	0.308
Cyp2j6	0.930	1.727	0.525	1.125	0.036	0.533	0.263
Pld1	0.070	0.328	0.013	0.112	0.058	0.818	0.212
Ptges	0.238	0.650	0.113	0.450	0.092	1.000	0.214
Ptges2	0.119	0.384	0.213	0.469	0.130	1.000	0.225
Ptges3l	0.042	0.201	0.025	0.224	0.574	1.000	0.081
Pla2r1	0.021	0.186	0.000	0.000	0.181	1.000	0.140
Pld2	0.329	0.700	0.413	0.706	0.395	1.000	0.119
Ptgs2	0.014	0.118	0.025	0.157	0.585	1.000	0.083
Pla2g4a	0.070	0.422	0.038	0.249	0.471	1.000	0.088
Cyp2c68	0.000	0.000	0.013	0.112	0.320	1.000	0.187
Cyp2j5	0.000	0.000	0.013	0.112	0.320	1.000	0.187
Cyp2j9	1.084	1.714	1.163	1.642	0.736	1.000	0.047
Cyp2j11	0.007	0.084	0.000	0.000	0.319	1.000	0.104
Cyp2j12	0.021	0.186	0.000	0.000	0.181	1.000	0.140
Cyp2j13	0.014	0.118	0.000	0.000	0.158	1.000	0.148

Supplementary Table 4: Single cell mRNA comparisons for mural cells

a. **Contractile machinery:**

See Supplementary Figure 3a-b

Gene name	Cortex Mean	Cortex SD	HC Mean	HC SD	p-value	Adj. p-value	Effect Size
Acta1	0.194	0.474	0.000	0.194	0.002	0.099	0.468
Ppp1r12c	1.661	2.541	0.550	1.111	0.004	0.206	0.492
Ppp1r9a	0.500	1.004	0.150	0.350	0.024	1.000	0.391
Ppp1r13b	0.161	0.606	0.000	0.161	0.040	1.000	0.305
Calm2	13.194	17.675	7.450	5.744	0.047	1.000	0.361
Ppp1r1b	0.081	0.329	0.000	0.081	0.058	1.000	0.281
Ppp1r26	0.081	0.375	0.000	0.081	0.096	1.000	0.246
Acta2	43.839	40.397	30.500	13.339	0.120	1.000	0.350
Des	0.581	0.984	1.000	-0.419	0.146	1.000	0.411
Tpm3	0.613	1.077	1.200	-0.587	0.153	1.000	0.472
Calm1	17.597	28.506	12.050	5.547	0.162	1.000	0.221
Ppp1r11	0.532	1.251	0.250	0.282	0.164	1.000	0.251
Ppp1r3b	0.048	0.282	0.000	0.048	0.182	1.000	0.196
Ppp1r3g	0.048	0.282	0.000	0.048	0.182	1.000	0.196
Ppp1r16b	0.371	0.707	0.200	0.171	0.188	1.000	0.264
Ppp1r2	0.597	1.273	0.350	0.247	0.240	1.000	0.215
Ppp1r18	0.016	0.127	0.100	-0.084	0.249	1.000	0.450
Calm3	1.323	2.209	0.900	0.423	0.274	1.000	0.210
Ppp1r3f	0.048	0.381	0.000	0.048	0.321	1.000	0.145
Ppp1r36	0.016	0.127	0.000	0.016	0.321	1.000	0.145
Ppp1r16a	0.016	0.127	0.000	0.016	0.321	1.000	0.145
Ppp1r13l	0.016	0.127	0.000	0.016	0.321	1.000	0.145
Mylk	2.677	3.018	3.400	-0.723	0.343	1.000	0.242
Ppp1ca	0.952	1.431	0.700	0.252	0.410	1.000	0.186
Ppp1r12b	0.081	0.329	0.200	-0.119	0.467	1.000	0.269
Ppp1r15a	2.306	3.687	2.800	-0.494	0.467	1.000	0.146
Vim	4.565	4.762	5.450	-0.885	0.512	1.000	0.181
Ppp1r10	0.161	0.413	0.250	-0.089	0.513	1.000	0.198
Ppp1r35	0.016	0.127	0.050	-0.034	0.525	1.000	0.218
Tpm4	1.484	1.725	1.700	-0.216	0.539	1.000	0.133
Ppp1r14c	0.065	0.248	0.100	-0.035	0.643	1.000	0.135
Ppp1r7	0.306	0.692	0.250	0.056	0.672	1.000	0.088
Tpm1	9.774	8.360	10.700	-0.926	0.683	1.000	0.109
Ppp1r14b	0.113	0.367	0.150	-0.037	0.696	1.000	0.101
Ppp1cb	1.500	1.990	1.650	-0.150	0.734	1.000	0.079
Ppp1r3d	0.065	0.307	0.100	-0.035	0.744	1.000	0.103
Ppp1r21	0.081	0.275	0.100	-0.019	0.804	1.000	0.068
Ppp1cc	0.226	0.612	0.250	-0.024	0.849	1.000	0.042
Ppp1r9b	0.177	0.426	0.200	-0.023	0.862	1.000	0.050
Ppp1r15b	0.274	0.705	0.250	0.024	0.874	1.000	0.036
Ppp1r3c	0.323	1.021	0.350	-0.027	0.891	1.000	0.029
Ppp1r1a	0.113	0.367	0.100	0.013	0.908	1.000	0.033
Ppp1r14a	0.210	0.517	0.200	0.010	0.932	1.000	0.020

Ppp1r12a	2.387	4.006	2.450	-0.063	0.935	1.000	0.017
Myh11	5.806	6.633	5.700	0.106	0.941	1.000	0.017
Tpm2	7.984	8.818	7.850	0.134	0.952	1.000	0.015
Ppp1r8	0.097	0.534	0.100	-0.003	0.973	1.000	0.007
Ppp1r2.ps3	0.048	0.216	0.050	-0.002	0.978	1.000	0.007

b. Ion channels:

See Figure 6g, and Supplementary Figure 3c-d

Gene name	Cortex Mean	Cortex SD	HC Mean	HC SD	p-value	Adj. p-value	Effect Size
Cacnb4	0.790	1.621	0.000	0.000	0.000	0.021	0.558
Cacng2	0.226	0.584	0.000	0.000	0.003	0.246	0.442
Kcnh3	0.161	0.451	0.000	0.000	0.007	0.455	0.410
Cacna1i	0.177	0.497	0.000	0.000	0.007	0.455	0.409
Kcnj4	0.194	0.568	0.000	0.000	0.009	0.637	0.390
Kcnc1	0.290	0.687	0.050	0.224	0.019	1.000	0.394
Cacna2d1	1.145	1.587	0.550	0.686	0.022	1.000	0.417
Cacng3	0.081	0.275	0.000	0.000	0.024	1.000	0.336
Kcnc4	0.145	0.507	0.000	0.000	0.028	1.000	0.328
Kcnh7	0.210	0.750	0.000	0.000	0.031	1.000	0.320
Cacna1a	1.000	1.708	0.400	0.754	0.032	1.000	0.391
Kcnb1	1.274	2.847	0.500	0.761	0.056	1.000	0.308
Kcnj2	0.081	0.329	0.000	0.000	0.058	1.000	0.281
Kcnj10	0.387	1.107	0.100	0.308	0.070	1.000	0.294
Kcnd3	0.048	0.216	0.000	0.000	0.083	1.000	0.256
Kcnn1	0.081	0.375	0.000	0.000	0.096	1.000	0.246
Cacna1e	0.726	1.935	1.650	2.368	0.125	1.000	0.452
Kcnk7	0.081	0.417	0.000	0.000	0.133	1.000	0.222
Kcnt1	0.387	0.930	0.150	0.489	0.146	1.000	0.280
Kcnf1	0.065	0.356	0.000	0.000	0.159	1.000	0.207
Kcnh4	0.032	0.178	0.000	0.000	0.159	1.000	0.207
Kcnq1	0.032	0.178	0.000	0.000	0.159	1.000	0.207
Kcnq5	0.032	0.178	0.000	0.000	0.159	1.000	0.207
Cacnb1	0.032	0.178	0.000	0.000	0.159	1.000	0.207
Kcnk13	0.000	0.000	0.100	0.308	0.163	1.000	0.667
Kcnj6	0.065	0.248	0.300	0.733	0.173	1.000	0.564
Cacna1h	1.274	2.189	0.750	1.209	0.181	1.000	0.262
Kcnq3	0.048	0.282	0.000	0.000	0.182	1.000	0.196
Kcna2	0.500	0.901	0.300	0.470	0.203	1.000	0.244
Kcnc3	0.194	0.538	0.450	0.826	0.205	1.000	0.414
Kcnh2	0.065	0.400	0.000	0.000	0.208	1.000	0.185
Kcnh5	0.065	0.400	0.000	0.000	0.208	1.000	0.185
Kcns2	0.065	0.400	0.000	0.000	0.208	1.000	0.185
Kcnj8	0.210	0.656	0.650	1.496	0.215	1.000	0.475
Kcnb2	0.016	0.127	0.100	0.308	0.249	1.000	0.450
Kcnc2	0.016	0.127	0.100	0.308	0.249	1.000	0.450
Kcnd2	0.210	0.604	0.100	0.308	0.291	1.000	0.200
Kcnma1	0.710	1.419	0.400	1.095	0.314	1.000	0.230

Kcnn4	0.016	0.127	0.000	0.000	0.321	1.000	0.145
Kcnu1	0.032	0.254	0.000	0.000	0.321	1.000	0.145
Kcnj13	0.016	0.127	0.000	0.000	0.321	1.000	0.145
Kcnj16	0.016	0.127	0.000	0.000	0.321	1.000	0.145
Kcnk6	0.016	0.127	0.000	0.000	0.321	1.000	0.145
Kcnk9	0.016	0.127	0.000	0.000	0.321	1.000	0.145
Kcnq4	0.016	0.127	0.000	0.000	0.321	1.000	0.145
Kcns3	0.032	0.254	0.000	0.000	0.321	1.000	0.145
Cacng5	0.016	0.127	0.000	0.000	0.321	1.000	0.145
Kcnj11	0.000	0.000	0.050	0.224	0.330	1.000	0.459
Kcna7	0.000	0.000	0.050	0.224	0.330	1.000	0.459
Cacng7	0.000	0.000	0.050	0.224	0.330	1.000	0.459
Cacng8	0.000	0.000	0.050	0.224	0.330	1.000	0.459
Cacna1f	0.000	0.000	0.050	0.224	0.330	1.000	0.459
Cacna2d2	0.000	0.000	0.100	0.447	0.330	1.000	0.459
Kcna1	0.516	1.067	0.300	0.923	0.387	1.000	0.209
Cacnb3	0.323	0.901	0.200	0.410	0.406	1.000	0.151
Cacnb2	0.065	0.307	0.200	0.696	0.408	1.000	0.313
Kcnv1	0.113	0.483	0.050	0.224	0.429	1.000	0.145
Kcnq2	0.339	0.788	0.200	0.696	0.458	1.000	0.181
Cacna1d	0.710	1.475	0.500	0.946	0.461	1.000	0.153
Cacna2d3	0.194	0.698	0.100	0.447	0.487	1.000	0.145
Kcna6	0.032	0.178	0.100	0.447	0.516	1.000	0.253
Cacna1c	0.194	0.596	0.300	0.657	0.524	1.000	0.174
Kcna5	0.032	0.254	0.100	0.447	0.525	1.000	0.218
Kcnk1	0.081	0.329	0.050	0.224	0.640	1.000	0.100
Kcnj12	0.210	0.517	0.150	0.489	0.643	1.000	0.117
Kcnt2	0.129	0.383	0.100	0.308	0.732	1.000	0.079
Cacna1g	0.194	0.507	0.150	0.489	0.734	1.000	0.087
Kcna4	0.032	0.178	0.050	0.224	0.749	1.000	0.093
Cacna1b	0.032	0.254	0.050	0.224	0.767	1.000	0.072
Kcnk3	0.177	0.497	0.200	0.410	0.840	1.000	0.047
Kcnn2	0.113	0.367	0.100	0.308	0.877	1.000	0.036
Kcnk2	0.097	0.469	0.100	0.308	0.972	1.000	0.007

c. **Neurovascular signalling pathways:**

Gene name	Cortex Mean	Cortex SD	HC Mean	HC SD	p-value	Adj. p-value	Effect Size
Cyp2j9	0.226	0.838	0.000	0.000	0.038	0.683	0.309
Grin2b	1.871	3.123	0.800	1.609	0.050	0.846	0.377
Nos1	0.000	0.000	0.100	0.308	0.163	1.000	0.667
Grin2c	0.452	2.400	0.100	0.447	0.277	1.000	0.167
Gucy1a2	0.823	1.124	0.600	0.681	0.291	1.000	0.215
Grin2d	0.016	0.127	0.000	0.000	0.321	1.000	0.145
Cyp2c50	0.016	0.127	0.000	0.000	0.321	1.000	0.145
Grin1	0.661	1.414	0.450	0.826	0.415	1.000	0.163
Grin2a	0.065	0.248	0.150	0.489	0.461	1.000	0.266
Gucy1b3	1.452	2.434	1.800	1.908	0.512	1.000	0.150
Gucy2g	0.016	0.127	0.050	0.224	0.525	1.000	0.218

Nos3	0.435	1.350	0.300	0.733	0.570	1.000	0.110
Cyp2j6	0.129	0.424	0.100	0.308	0.741	1.000	0.073
Ptges3l	0.355	1.010	0.300	0.733	0.793	1.000	0.058
Ptges	0.065	0.400	0.050	0.224	0.839	1.000	0.040
Gucy1a3	1.597	2.336	1.500	2.090	0.862	1.000	0.042
Ptges3	0.629	0.962	0.650	1.040	0.937	1.000	0.021
Ptges2	0.048	0.216	0.050	0.224	0.978	1.000	0.007

Supplementary Table 5: Single cell mRNA comparisons for endothelial cells

a. **Ion channels:**

See Figure 6h

Gene name	Cortex Mean	Cortex SD	HC Mean	HC SD	p-value	Adj. p-value	Effect Size
Kcnj10	0.200	0.508	0.000	0.000	0.000	0.002	0.408
Kcnj2	0.184	0.530	0.000	0.000	0.000	0.012	0.360
Kcna1	0.376	1.148	0.000	0.000	0.000	0.027	0.339
Kcnh3	0.104	0.377	0.000	0.000	0.003	0.185	0.286
Cacnb3	0.080	0.301	0.000	0.000	0.004	0.253	0.276
Kcnk1	0.104	0.398	0.000	0.000	0.004	0.294	0.271
Kcnk3	0.056	0.231	0.000	0.000	0.008	0.534	0.251
Cacna1c	0.216	0.903	0.000	0.000	0.009	0.588	0.248
Cacng3	0.064	0.277	0.000	0.000	0.011	0.737	0.240
Cacnb4	0.720	2.684	0.100	0.316	0.019	1.000	0.239
Kcnj8	0.552	2.653	0.000	0.000	0.022	1.000	0.215
Kcnj12	0.040	0.197	0.000	0.000	0.025	1.000	0.211
Kcnh5	0.072	0.363	0.000	0.000	0.028	1.000	0.205
Kcna6	0.080	0.433	0.000	0.000	0.041	1.000	0.192
Kcnv1	0.080	0.433	0.000	0.000	0.041	1.000	0.192
Kcnma1	0.824	2.229	0.300	0.483	0.042	1.000	0.243
Kcnk7	0.032	0.177	0.000	0.000	0.045	1.000	0.188
Kcnc4	0.024	0.154	0.000	0.000	0.083	1.000	0.162
Kcnf1	0.048	0.307	0.000	0.000	0.083	1.000	0.162
Kcns2	0.024	0.154	0.000	0.000	0.083	1.000	0.162
Kcnn2	0.032	0.218	0.000	0.000	0.103	1.000	0.152
Kcnt2	0.032	0.218	0.000	0.000	0.103	1.000	0.152
Kcnj14	0.072	0.495	0.000	0.000	0.106	1.000	0.151
Kcnj4	0.144	0.998	0.000	0.000	0.109	1.000	0.149
Kcnj11	0.072	0.511	0.000	0.000	0.118	1.000	0.146
Cacna1i	0.040	0.295	0.000	0.000	0.132	1.000	0.140
Kcnn3	0.016	0.126	0.000	0.000	0.158	1.000	0.132
Kcnj16	0.016	0.126	0.000	0.000	0.158	1.000	0.132
Kcnj3	0.016	0.126	0.000	0.000	0.158	1.000	0.132
Kcnk10	0.016	0.126	0.000	0.000	0.158	1.000	0.132
Kcnk13	0.016	0.126	0.000	0.000	0.158	1.000	0.132
Kcnd1	0.016	0.126	0.000	0.000	0.158	1.000	0.132
Kcng2	0.016	0.126	0.000	0.000	0.158	1.000	0.132
Kcnh4	0.016	0.126	0.000	0.000	0.158	1.000	0.132
Kcnn1	0.024	0.199	0.000	0.000	0.181	1.000	0.125
Kcnu1	0.024	0.199	0.000	0.000	0.181	1.000	0.125
Cacna1e	0.552	1.316	2.500	4.275	0.184	1.000	1.153
Kcnc3	0.136	0.428	0.700	1.252	0.189	1.000	1.072
Kcnk2	0.128	0.457	0.400	0.699	0.255	1.000	0.569
Kcnd2	0.176	0.597	0.900	1.912	0.263	1.000	0.951
Cacng2	0.240	0.846	0.100	0.316	0.276	1.000	0.170
Kcnj15	0.016	0.179	0.000	0.000	0.319	1.000	0.093
Kcnj1	0.008	0.089	0.000	0.000	0.319	1.000	0.093

Kcnk5	0.016	0.179	0.000	0.000	0.319	1.000	0.093
Kcna5	0.016	0.179	0.000	0.000	0.319	1.000	0.093
Kcng1	0.008	0.089	0.000	0.000	0.319	1.000	0.093
Kcng1.1	0.008	0.089	0.000	0.000	0.319	1.000	0.093
Kcnq4	0.016	0.179	0.000	0.000	0.319	1.000	0.093
Kcns1	0.016	0.179	0.000	0.000	0.319	1.000	0.093
Cacng4	0.008	0.089	0.000	0.000	0.319	1.000	0.093
Cacng5	0.040	0.447	0.000	0.000	0.319	1.000	0.093
Cacng8	0.008	0.089	0.000	0.000	0.319	1.000	0.093
Cacna2d2	0.008	0.089	0.000	0.000	0.319	1.000	0.093
Cacna1b	0.008	0.089	0.100	0.316	0.383	1.000	0.771
Cacna1d	0.584	1.199	1.200	2.150	0.393	1.000	0.479
Cacna1h	0.208	0.873	0.100	0.316	0.404	1.000	0.127
Cacna1a	0.664	1.047	1.100	1.595	0.416	1.000	0.399
Kcnb2	0.016	0.126	0.100	0.316	0.425	1.000	0.572
Kcna4	0.024	0.154	0.100	0.316	0.470	1.000	0.448
Kcnd3	0.024	0.199	0.100	0.316	0.472	1.000	0.363
Kcnq2	0.240	0.865	0.500	1.080	0.475	1.000	0.295
Kcnc1	0.184	0.640	0.100	0.316	0.477	1.000	0.135
Cacna2d3	0.040	0.295	0.100	0.316	0.574	1.000	0.202
Cacna2d1	0.632	1.692	1.000	2.000	0.584	1.000	0.215
Cacna1g	0.120	0.548	0.200	0.422	0.584	1.000	0.148
Kcnq3	0.048	0.333	0.100	0.316	0.628	1.000	0.157
Kcnj6	0.144	0.564	0.100	0.316	0.700	1.000	0.080
Kcnj9	0.288	0.914	0.400	0.966	0.730	1.000	0.122
Kcnt1	0.128	0.508	0.200	0.632	0.733	1.000	0.139
Kcnq1	0.136	0.446	0.100	0.316	0.744	1.000	0.082
Kcnc2	0.136	0.497	0.100	0.316	0.748	1.000	0.074
Kcnh7	0.128	0.457	0.100	0.316	0.800	1.000	0.062
Kcna2	0.432	1.180	0.400	0.516	0.871	1.000	0.028
Kcnb1	0.768	2.342	0.700	1.252	0.881	1.000	0.030
Kcnq5	0.112	0.571	0.100	0.316	0.916	1.000	0.022
Cacnb1	0.104	0.521	0.100	0.316	0.972	1.000	0.008

b. **Neurovascular signalling pathways:**

See Figure 6i, Supplementary Figures 3e-f

**Gene name**	**Cortex Mean**	**Cortex SD**	**HC Mean**	**HC SD**	**p-value**	**Adj. p-value**	**Effect Size**
Gucy1a2	0.240	0.677	0.000	0.000	0.000	0.002	0.367
Grin2c	0.560	1.701	0.000	0.000	0.000	0.005	0.341
Ptges	0.176	0.540	0.000	0.000	0.000	0.005	0.338
Cyp2j6	0.168	0.632	0.000	0.000	0.004	0.046	0.275
Ptges2	0.056	0.231	0.000	0.000	0.008	0.092	0.251
Gucy1a3	0.264	1.212	0.000	0.000	0.016	0.180	0.226
Gucy1b3	0.408	1.232	0.100	0.316	0.045	0.450	0.258
Ptges3l	0.088	0.492	0.000	0.000	0.048	0.450	0.185
Nos1	0.016	0.126	0.000	0.000	0.158	1.000	0.132
Grin2a	0.048	0.307	0.500	0.972	0.176	1.000	1.159

Ptges3	0.432	0.892	0.900	0.994	0.179	1.000	0.520
Nos3	0.768	1.130	0.500	0.707	0.295	1.000	0.242
Cyp2j5	0.008	0.089	0.000	0.000	0.319	1.000	0.093
Cyp2j9	0.008	0.089	0.000	0.000	0.319	1.000	0.093
Grin2b	1.232	3.290	2.900	5.259	0.348	1.000	0.482
Grin1	0.456	1.428	0.900	1.912	0.489	1.000	0.303

Supplementary Table 6: Comparing average number of mRNA transcripts across the vascular beds of HC and neocortex

Data from Figure/ Table	Mean	Standard Deviation	Test	Test Statistic	95% Confidence Interval	Degrees of Freedom	P value
**Supplementary Data Table 4a: Mural Cells, Contractile Machinery**	HC: 2.0750 Ctx: 2.5692	HC: 5.0452 Ctx: 7.0453	Two-tailed unpaired t-test, unequal variances	t=0.3951	-1.9925 to 2.9810	df=85.17	p=0.6937
**Supplementary Data Table 4b: Mural Cells, Ion Channels**	HC: 0.1389 Ctx: 0.1956	HC: 0.2483 Ctx: 0.2933	Two-tailed unpaired t-test, unequal variances	t=1.2514	-0.0329 to 0.1462	df=138.23	p=0.2129
**Supplementary Data Table 4c: Mural Cells NV signalling**	HC: 0.3889 Ctx: 0.4919	HC: 0.5220 Ctx: 0.5896	Two-tailed unpaired t-test, unequal variances	t=0.5552	-0.2744 to 0.4805	df=33.5085	p=0.5825
**Supplementary Data Table 5a: Endothelial Cells, Ion Channels**	HC: 0.1592 Ctx: 0.1355	HC: 0.3799 Ctx: 0.1893	Two-tailed unpaired t-test, unequal variances	t=-0.4875	-0.1202 to 0.0728	df=110.09	p=0.6269
**Supplementary Data Table 5b: Endothelial Cells, NV signalling**	HC: 0.3625 Ctx: 0.3080	HC: 0.7500 Ctx: 0.3331	Two-tailed unpaired t-test, unequal variances	t=-0.2656	-0.4815 to 0.3725	df=20.6966	p=0.7931

Supplementary Table 7: Artery/Vein Ratio in CA1 and V1

Region	Number Animals	Number Stacks	Mean # Arteries	SD Arteries	Mean # Veins	SD Veins	Artery/Vein Ratio
**HC**	3	5	1.2	0.45	2.2	0.45	0.53
**V1**	8	8	1.25	0.46	2	0.76	0.69
**Compare Groups:**							
t(11)=0.65, p=0.5319							

Supplementary References

1. Zeisel, A. *et al.* Molecular Architecture of the Mouse Nervous System. *Cell* **174**, 999-1014.e22 (2018).
