# Supplementary material for: Neurovascular coupling and oxygenation are decreased in hippocampus compared to neocortex because of microvascular differences
Source: Nat Commun. 2021 May 27;12:3190. doi: 10.1038/s41467-021-23508-y (PMC8160329; doi:10.1038/s41467-021-23508-y)
Supplement: Supplementary file 3 — Reporting summary [file 41467_2021_23508_MOESM3_ESM.pdf]

nature research

Corresponding author(s): Catherine Hall

Last updated by author(s): 09/03/2021

Reporting Summary

Nature Research wishes to improve the reproducibility of the work that we publish. This form provides structure for consistency and transparency in reporting. For further information on Nature Research policies, see Authors & Referees and the Editorial Policy Checklist .

Statistics

For all statistical analyses, confirm that the following items are present in the figure legend, table legend, main text, or Methods section.

n/a	Confirmed	
☐	☒	The exact sample size (*n*) for each experimental group/condition, given as a discrete number and unit of measurement
☐	☒	A statement on whether measurements were taken from distinct samples or whether the same sample was measured repeatedly
☐	☒	The statistical test(s) used AND whether they are one- or two-sided<br>*Only common tests should be described solely by name; describe more complex techniques in the Methods section.*
☐	☒	A description of all covariates tested
☐	☒	A description of any assumptions or corrections, such as tests of normality and adjustment for multiple comparisons
☐	☒	A full description of the statistical parameters including central tendency (e.g. means) or other basic estimates (e.g. regression coefficient) AND variation (e.g. standard deviation) or associated estimates of uncertainty (e.g. confidence intervals)
☐	☒	For null hypothesis testing, the test statistic (e.g. *F*, *t*, *r*) with confidence intervals, effect sizes, degrees of freedom and *P* value noted<br>*Give P values as exact values whenever suitable.*
☒	☐	For Bayesian analysis, information on the choice of priors and Markov chain Monte Carlo settings
☒	☐	For hierarchical and complex designs, identification of the appropriate level for tests and full reporting of outcomes
☐	☒	Estimates of effect sizes (e.g. Cohen's *d*, Pearson's *r*), indicating how they were calculated

Our web collection on statistics for biologists contains articles on many of the points above.

Software and code

Policy information about availability of computer code

Data collection	SciScan v1.2.1 (Scientifica) was used to collect 2P data, Leica SP8 confocal data was collected using LAS X software (details: https://www.leica-microsystems.com/products/microscope-software/p/leica-las-x-ls/), and haemoglobin spectroscopy data was collected in Moor instruments VMS V4.0 software. Raw tif image files were visualised in ImageJ FIJI and/or MATLAB 2019b. In MATLAB, the cellSort package (v1.4) was used to extract neuronal calcium traces from tif files.
Data analysis	Custom code used to analyse the data is available on the github repository: https://github.com/BrainEnergyLab/HCvsV1_NVC_Manuscript

For manuscripts utilizing custom algorithms or software that are central to the research but not yet described in published literature, software must be made available to editors/reviewers. We strongly encourage code deposition in a community repository (e.g. GitHub). See the Nature Research guidelines for submitting code & software for further information.

Data

Policy information about availability of data

All manuscripts must include a data availability statement. This statement should provide the following information, where applicable:

- Accession codes, unique identifiers, or web links for publicly available datasets
- A list of figures that have associated raw data
- A description of any restrictions on data availability

The mRNA data presented in Figure 4 and Supplementary Figure 8 were taken from: http://linnarssonlab.org/cortex/. The source data used for the figure plots in Figures 2, 3, 5, 6, 7, and Supplementary Figures 1-7 & 9-10 are provided as a Source Data File (Microsoft Excel document). For all main figures, and the supplementary figures with unique datasets, the extracted traces from the raw image files are available as .xlxs and .mat files on Figshare: 10.25377/sussex.10289153. The raw image files are stored in our Dropbox due to their large size, and are available from the corresponding author upon request.

Field-specific reporting

Please select the one below that is the best fit for your research. If you are not sure, read the appropriate sections before making your selection.

- [x] Life sciences
- [ ] Behavioural & social sciences
- [ ] Ecological, evolutionary & environmental sciences

For a reference copy of the document with all sections, see nature.com/documents/nr-reporting-summary-flat.pdf

Life sciences study design

All studies must disclose on these points even when the disclosure is negative.

Sample size	We were not able to conduct prior power analyses to predict sample sizes for these experiments, as we did not know what effect size to expect. Considering our main finding (Figure 3) of a difference in dilation size between HC and V1, for a medium effect size of 0.5, at the outset we expected to have to record 63 dilation events in each region to detect an effect with alpha = 0.05 and 80% power. However, we also wanted to record from at least 4 mice per condition, and several vessels per mouse, to minimise the effect of any inter-animal differences. To achieve this minimum requirement we performed surgeries on more mice in case of losses in recovery, but in fact in the imaging sessions planned we were able to record from 40-41 vessels in 6-7 mice per region (at least 4 animals per group contributed at least 4 vessels to the dataset). This resulted in 120 dilation events in HC and 313 in V1, meaning that our effect size for the difference between the dilation size in HC and V1 was 0.39, and our power to detect this at alpha = 0.05 was 95%.
Data exclusions	No full data recordings were excluded from the analysis once they had met the criteria to be preprocessed and extracted (i.e. showed a clear signal above noise in the raw image files). After extracting the two-photon data, some detected neuronal calcium peaks were removed if: the peaks were >2 order of magnitudes bigger than other peaks in the recording, and if upon rechecking the raw files this large magnitude shift could be attributed to signal loss due to motion artefacts (e.g. changes in z depth).
Replication	Data was taken from multiple recording sessions and multiple animals (53 animals were used across 4 imaging techniques: two-photon, oxyprobe, behaviour, confocal imaging of fixed tissue). All data included in the manuscript represented information collected from at least 3 animals per brain region. All experimental recordings were independent (as individual vessels or cells were not tracked longitudinally over time, or across multiple imaging techniques). Experimental conclusions are strengthened by replication across multiple experimental techniques where possible, e.g. haemoglobin spectroscopy findings supported the results observed from two-photon microscopy (vascular reactivity) and confocal images (vascular density).
Randomization	Randomization was not applicable to our study because the mice used for both brain region groups came from the same breeding colonies, and were balanced for age and sex, reducing the risk of a selection bias.
Blinding	Investigators were not blinded during data collection, as the surgical cranial windows for each group (hippocampus or cortex) were visibly very distinct. The investigators were blinded to animal ID/brain region for any data analyses which involved manual scoring (e.g. categorising pericytes or vessel lengths from z stacks). The investigators were not blinded to brain region per se for in vivo time series analyses, however the analysis was entirely automated (so did not require manual input from the experimenter).

Reporting for specific materials, systems and methods

We require information from authors about some types of materials, experimental systems and methods used in many studies. Here, indicate whether each material, system or method listed is relevant to your study. If you are not sure if a list item applies to your research, read the appropriate section before selecting a response.

Materials & experimental systems

n/a	Involved in the study
[ ]	[x] Antibodies
[x]	[ ] Eukaryotic cell lines
[x]	[ ] Palaeontology
[ ]	[x] Animals and other organisms
[x]	[ ] Human research participants
[x]	[ ] Clinical data

Methods

n/a	Involved in the study
[x]	[ ] ChIP-seq
[x]	[ ] Flow cytometry
[x]	[ ] MRI-based neuroimaging

Antibodies

Antibodies used	Chicken anti-GFAP primary antibody (Abcam, ab4674, 1:500 dilution), rabbit anti-Iba1 (WAKO, 019-19741, 1:600 dilution), Alexa 647 goat anti-chicken (Abcam, ab150171, 1:500 dilution), Alexa 647 goat anti-rabbit (Abcam, ab150079, 1:500 dilution)
Validation	Primary Antibodies:<br><br>1. Chicken anti-GFAP (1:500, Abcam ab4674).<br>Details: Chicken polyclonal to GFAP

Isotype: IgY
Tested application: IHC (PFA fixed), IHC-FrFl, ICC, IHC-P, WB
Validation species: Mouse, Rat
References: https://www.abcam.com/gfap-antibody-ab4674.html

2. Rabbit anti-Iba1 (1:600, WAKO, 019-19741)
Details: Rabbit polyclonal to Iba1a
Antigen: Synthetic peptide (C-terminal of Iba1)
Tested application: IHC (PFA fixed and frozen)
Validation species: Mouse, rat, human
References: https://labchem-wako.fujifilm.com/us/category/01213.html

Secondary antibodies:

1. Alexa 647 goat anti-chicken (1:500, Abcam, ab150171)
Details: Goat polyclonal Secondary Antibody to Chicken IgY - H&L (Alexa Fluor® 647)
Conjugation: Alexa Fluor® 647. Ex: 652nm, Em: 668nm
Host species: Goat
Isotype: IgG
Suitable for: IHC-Fr, ICC/IF, ELISA, IHC-P, Flow Cyt
Reference: https://www.abcam.com/goat-chicken-igy-hl-alexa-fluor-647-ab150171.html

2. Alexa 647 goat anti-rabbit (1:500, Abcam, ab150079)
Details: Goat polyclonal Secondary Antibody to Rabbit IgG - H&L (Alexa Fluor® 647)
Conjugation: Alexa Fluor® 647. Ex: 652nm, Em: 668nm
Host species: Goat
Isotype: IgG
Suitable for: IHC-Fr, ICC/IF, ELISA, IHC-P, Flow Cyt
Reference: https://www.abcam.com/goat-rabbit-igg-hl-alexa-fluor-647-ab150079.html

Animals and other organisms

Policy information about studies involving animals; ARRIVE guidelines recommended for reporting animal research

Laboratory animals	All experiments used mice with a C57BL/6J background of either sex, which were either wild types (6 in total, 4 males, 2 females) or expressed GCaMP6f under the control of the Thy1 promoter (C57BL/6J-Tg(Thy1-GCaMP6f)GP5.5Dkim/J 13; 30 in total, 15 males, 15 females) and/or DsRed under the control of the NG2 promoter (NG2DsRedBAC 49; 17 total, 9 males, 8 females).
Wild animals	No wild animals were used in this study
Field-collected samples	No field collected samples were used in this study
Ethics oversight	All experimental procedures were approved by the UK Home Office, in accordance with the 1986 Animal (Scientific Procedures) Act and the Animal Welfare Ethical Review Board (AWERB) at the University of Sussex.

Note that full information on the approval of the study protocol must also be provided in the manuscript.
