# Supplementary material for: Neurovascular coupling and oxygenation are decreased in hippocampus compared to neocortex because of microvascular differences
Source: Nat Commun. 2021 May 27;12:3190. doi: 10.1038/s41467-021-23508-y (PMC8160329; doi:10.1038/s41467-021-23508-y)
Supplement: Supplementary file 4 — Source Data [file 41467_2021_23508_MOESM4_ESM.zip › Shawetal_StatisticsReports_March21.pdf]

Statistics Reports (SR1-12)

The following tables report the mean, standard deviation, statistical test, test statistics, degrees of freedom and p-value for each statistical test reported in the main manuscript or supplementary figures, where not already reported in Supplementary Tables 1-6.

SR1: Figure 2: Baseline haemodynamics in HC and V1 (comparison of response sizes)

All two group comparisons were first subject to an F test to compare variances, those with equal variances were subject to independent sample t-tests, and those which showed significant differences between groups Mann-Whitney U tests.

Figure Label	Mean	Standard Deviation	Test	Test Statistic	95% Confidence Interval	Degrees of Freedom	P value
**2a ($CMRO_2$)**	HC: 196.1<br>V1: 185.7	HC: 61.94<br>V1: 45.25	Two-tailed unpaired t-test	t=0.4042	-64.54 to 43.87	16	p=0.6914
**2b (CBF)**	HC: 282.3<br>V1: 404.2	HC: 93.82<br>V1: 97.39	Two-tailed unpaired t-test	t=2.706	26.44 to 217.5	16	p=0.0156*
**2c (SO_2)**	HC: 30.13<br>V1: 53.84	HC: 3.477<br>V1: 5.372	Two-tailed unpaired t-test	t=11.11	19.19 to 28.23	16	p=6.2E-9*
**2e (cap. density)**	HC: 1.494<br>V1: 2.802	HC: 0.2911<br>V1: 0.7169	Two-tailed unpaired t-test	t=4.116	0.5891 to 2.027	9	p=0.0026*
**2f (linescan diameters)**	HC: 4.891<br>V1: 5.237	HC: 1.333<br>V1: 1.024	Two-tailed Welch's t-test	t=1.519	-0.1057 to 0.7982	107	p=0.1318
**2h (linescan haematocrit)**	HC: 43.42<br>V1: 47.48	HC: 9.785<br>V1: 9.677	Two-tailed unpaired t-test	t=2.180	0.3961 to 7.760	107	p=0.0314*
**2i (linescan flux)**	HC: 76.78<br>V1: 94.85	HC: 34.68<br>V1: 44.26	Two-tailed Mann-Whitney U test	u= 1125	1.646 to 29.82	107	p=0.0289*
**2j (linescan velocity)**	HC: 1.07<br>V1: 2.43	HC: 0.91<br>V1: 2.00	Two-tailed Mann-Whitney U test	u=728	0.439 to 1.45	107	p=4.5E-6*

SR2a: Figure 3: Vessel responses to local neuronal calcium events (comparison of response frequencies)

Figure Label	Test	Responsive	Non-Responsive	X-squared	Degrees of Freedom	P value
**3c (vessel responses)**	Pearson's Chi-squared test, with post-hoc pairwise comparisons	HC real: 120, V1 real: 313	HC real: 659, V1 real: 925	27.092	1	p<0.0001*

SR2b: Figure 3: Vessel responses to local neuronal calcium events (comparison of response sizes)

All two group comparisons were first subject to an F test to compare variances, those with equal variances were subject to independent sample t-tests, and those which showed significant differences between groups Mann-Whitney U tests.

Figure Label	Mean	Standard Deviation	Test	Test Statistic	95% Confidence Interval	Degrees of Freedom	P value
**Vessel diameters**	HC: 9.273 V1: 8.323	HC: 4.089 V1: 4.050	Two-tailed unpaired t-test	t=1.086	-2.688 to 0.7884	85	p=0.2803
**3g (calcium peaks, all)**	HC: 0.8096, V1: 0.9297	HC: 1.784, V1: 2.876	Two-tailed Mann-Whitney U test	u=430106	-0.08761 to -0.02981	2015	p=4.3E-5*
**3h (diameter peaks, all)**	HC: 0.0189, V1: 0.02496	HC: 0.02218, V1: 0.02997	Two-tailed Mann-Whitney U test	u=408192	0.002513 to 0.005181	2012	p=6.9E-9*
**3i (diameter, shuffled)**	HC: 0.004, V1: 0.004	HC: 0.0040, V1: 0.0033	Two-tailed Mann-Whitney U test	u=464171	-5.405e-005 to 0.0003297	2015	p=0.1569
**3l (calcium peaks, responsive)**	HC: 0.829, V1: 0.721	HC: 1.0428, V1: 1.0151	Two-tailed Mann-Whitney U test	u=15623	-0.1690 to -0.02528	431	p=0.0068*
**3m (diameter peaks, responsive)**	HC: 0.033, V1: 0.049	HC: 0.0359, V1: 0.0424	Two-tailed Mann-Whitney U test	u=12629	0.008046 to 0.01741	431	p=1.3E-7*
**3n (NVC_{index}, responsive)**	HC: 0.094, V1: 0.202	HC: 0.1492, V1: 0.2281	Two-tailed Mann-Whitney U test	u=12901	0.02510 to 0.07131	431	p=4.6E-7*

Figure 4: Vasodilatory second messenger pathways in HC and cortex
See Supplementary Data Tables 1-3.

SR3: Figure 5: Wide-field neuronal activity patterns

All two group comparisons were first subject to an F test to compare variances, those with equal variances were subject to independent sample t-tests, and those which showed significant differences between groups Mann-Whitney U tests.

Figure Label	Mean	Standard Deviation	Test	Test Statistic	95% Confidence Interval	Degrees of Freedom	P value
**5c (correlation)**	HC: 0.017 V1: 0.0336	HC: 0.0168 V1: 0.0319	Two-tailed Mann-Whitney U test	u=63	-0.001405 to 0.02988	27	p=0.1507
**5d (peak size)**	HC: 0.528 V1: 0.6598	HC: 0.3155 V1: 0.2142	Two-tailed unpaired t-test	t=1.338	-0.07043 to 0.3344	27	p=0.1921
**5h ($CMRO_2$)**	HC: median-4.886 V1: median-6.556	HC: 13.1863 V1: 4.5496	Two-tailed Mann-Whitney U test	u=355670	-1.302 to 0.4390	1920	p=4.1e-18*
**5j (Hbt)**	HC: median-2.213 V1: median-3.435	HC: 1.4633 V1: 3.4391	Two-tailed Mann-Whitney U test	t=16.53	1.780 to 2.259	1920	p=1.8E-44*
**5k (NVC_{index})**	HC: median-0.4492, V1: median-0.5509	HC: 1.8536, V1: 0.7475	Two-tailed Mann-Whitney U test	u=419850	0.7287 to 1.072	1920	p=6.8e-4*

SR4a: Figure 6: Vascular morphology across brain regions (comparison of vessel sizes and ISDs)

All two group comparisons were first subject to an F test to compare variances, those with equal variances were subject to independent sample t-tests, and those which showed significant differences between groups Mann-Whitney U tests.

Figure Label	Mean	Standard Deviation	Test	Test Statistic	95% Confidence Interval	Degrees of Freedom	P value
**6d (arteriole branches length)**	HC: 152.0 V1: 156.9	HC: 102.2 V1: 100.5	Two-tailed Mann-Whitney U test	u=291	108.8 to 198.4	47	p=0.8662
**6d (arteriole branches diameter)**	HC: 18.72 V1: 21.52	HC: 7.60 V1: 9.093	Two-tailed Mann-Whitney U test	u=258	15.51 to 25.27	47	p=0.4070
**6e (last arteriole branch length)**	HC: 97.78 V1: 135.5	HC: 54.79 V1: 81.45	Two-tailed unpaired t-test	t=1.036	-45.61 to 66.97	13	p=0.3189
**6e (last arteriole branch diameter)**	HC: 11.56 V1: 13.93	HC: 2.034 V1: 1.990	Two-tailed unpaired t-test	t=2.282	-1.880 to 6.150	13	p=0.04*
**6f (precapillary arteriole branches length)**	HC: 55.94 V1: 65.74	HC: 13.02 V1: 22.02	Two-tailed unpaired t-test	t=1.195	-7.493 to 27.08	17	p=0.2484
**6f (precapillary arteriole branches diameter)**	HC: 8.065 V1: 7.313	HC: 0.8158 V1: 1.916	Two-tailed unpaired t-test	t=1.134	-2.149 to 0.6463	17	p=0.2724
**6g (venule branches length)**	HC: 164.8 V1: 148.0	HC: 121.0 V1: 95.05	Two-tailed unpaired t-test	t=0.3992	-103.8 to 70.06	26	p=0.6930
**6g (venule branches diameter)**	HC: 23.42 V1: 26.74	HC: 10.65 V1: 7.852	Two-tailed unpaired t-test	t=0.9088	-4.188 to 10.83	26	p=0.3718
**6h (pericyte ISD)**	HC: 103.9 V1: 86.21	HC: 62.14 V1: 40.79	Two-tailed Mann-Whitney U test	u=4618	-22.59 to -0.4441	212	p=0.0415*

SR4b: Figure 6: Pericyte morphology across brain regions (comparison of frequencies of cell types)

Figure Label	Test	Counts	X-squared	Degrees of Freedom	P value
**6j (mural cell type)**	Pearson's Chi-squared test	HC: EP 15, MP 172, TSP 98 V1: EP 19, MP 206, TSP 154	2.7205	5	p=0.256593

SR4c: Figure 6: Vascular morphology across brain regions (comparison of cell lengths and diameters across regions)

We ran a multifactorial ANOVA on the vessel diameters and cell lengths presented in figure 6, however these were not presented in the figure as the ANOVAs assumption of equal variance between groups was violated.

Figure Label	Mean	Standard Deviation	Test	Test Statistic	Type III Sum of Squares	Degrees of Freedom	P value
**6k (confocal, diameters)**	HC: 3.4304 V1: 3.5712	HC: 1.9664 V1: 1.7842	Multifactorial ANOVA to compare region, cell type and region * cell type interaction	Region: F=0.064, Cell Type: F=142.649, Interaction: F=1.281	Region: 0.115, Cell Type: 514.172, Interaction: 4.618	Region: df=1, Cell Type: df=2, Interaction: df=2	Region: p=0.801, Cell Type: p=3.4E-52*, Interaction: p=0.278
**6l (confocal, cell lengths)**	HC: 103.8203 V1: 86.4535	HC: 42.93932 V1: 33.69613	Multifactorial ANOVA to compare region, cell type and region * cell type interaction	Region: F=12.525, Cell Type: F=43.651, Interaction: F=3.418	Region: 14272.770, Cell Type: 99480.056, Interaction: 7790.044	Region: df=1, Cell Type: df=2, Interaction: df=2	Region: p=0.0004*, Cell Type: p=1.6E-18*, Interaction: p=0.033*

SR4d: Figure 6: Vascular morphology across brain regions (general comparison of regional cell lengths and diameters for unequal variances)

The distribution of vessel diameters (p=0.02) and cell lengths (p<0.001) were unequal across brain regions (independent samples Mann-Whitney U test), meaning the variance differed across groups. Therefore, we also ran multiple one-way ANOVA tests using Welch's post-hoc comparison, which also demonstrated significant effects of cell type on diameter and of region and cell type on cell length.

Figure Label	Test	Test Statistic	Sum of Squares	Degrees of Freedom	P value
**6k (confocal, diameters)**	Welch statistic	Region: F=0.031 Cell Type: F=142.425	Region: 0.080, Cell Type: 513.382	662	Region: p=0.866, Cell Type: p=1.6E-30*
**6l (confocal, cell lengths)**	Welch statistic	Region: F=37.066 Cell Type: F=35.618	Region: 47649.155, Cell Type: 87426.439	662	Region: p=7.7E-9*, Cell Type: p=5.7E-36*

SR4e: Figure 6: Vascular morphology across brain regions (comparison of regional cell lengths and diameters within cell types for unequal variances)

In order to assess which cell types showed regional differences in our data, we also conducted multiple Mann-Whitney tests to compare means between regions for diameters and cell lengths of EP, MP and TSP, with Bonferroni correction applied to account for these multiple comparisons.

Figure Label	Mean	Standard Deviation	Test	Degrees of Freedom	P value
**6k (confocal, diameters)**	HC: EP-6.86, MP-3.36, TSP-2.50<br>V1: EP-6.75, MP-3.30, TSP-2.79	HC: EP-1.36, MP-1.91, TSP-0.79<br>V1: EP-1.37, MP-1.35, TSP-0.67	Multiple two-tailed Mann-Whitney U test, with Bonferroni correction	EP: 32<br>MP: 376<br>TSP: 250	EP: p=0.567 (before correction: 0.567)<br>MP: p=0.209 (before correction: 0.105)<br>TSP: p=2.65E-4* (before correction: 8.8E-5)
**6l (confocal, cell lengths)**	HC: EP-62.15, MP-102.1, TSP-125.75<br>V1: EP-57.11, MP-88.28, TSP-98.85	HC: EP-13.46, MP-38.53, TSP-39.41<br>V1: EP-10.54, MP-29.06, TSP-33.1	Multiple two-tailed Mann-Whitney U test, with Bonferroni correction	EP: 32<br>MP: 376<br>TSP: 250	EP: p=0.315 (before correction: 0.315)<br>MP: p=2.52E-4* (before correction: 1.26E-4)<br>TSP: p=2.07E-7* (before correction: 6.9E-8)

SR5a: Supplementary Figure 1: Vessel responses to local neuronal calcium events in capillaries taken by linescan recordings (comparison of ROI type frequencies)

Figure Label	Test	Counts	X-squared	Degrees of Freedom	P value
**SD 1b (linescan diameter)**	Pearson's Chi-squared test, 2x2 contingency table	HC: R: 81, NR: 365, V1: R: 109, NR: 291	9.487	1	p=0.0017*
**SD 1e (linescan RBCV)**	Pearson's Chi-squared test, 2x2 contingency table	HC: R: 126, NR: 212, V1: R: 107, NR: 198	0.335	1	p=0.5629

SR5b: Supplementary Figure 1: Vessel responses to local neuronal calcium events in capillaries (comparison of linescan velocity and dilation traces in responsive vessels)

All two group comparisons of linescan data through vessels and nearby neurons were first subject to an F test to compare variances, those with equal variances were subject to independent sample t-tests, and those which showed significant differences between groups Mann-Whitney U tests.

Figure Label	Mean	Standard Deviation	Test	95% Confidence Interval	Degrees of Freedom	P value
**Vessel diameters**	HC: 4.684 V1: 5.122	HC: 0.8425 V1: 1.222	Two-tailed unpaired t-test	-0.4570 to 1.333	23	0.3219
**SD 1d, 1e (linescan diameter)**	HC: 0.0153, V1: 0.0147	HC: 0.016, V1: 0.022	Two-tailed unpaired Mann-Whitney U test	-0.004675 to 0.0004332	188	p=0.1067
**SD 1f, 1g (linescan RBCV)**	HC: 0.1796, V1: 0.2248	HC: 0.1260, V1: 0.2079	Two-tailed unpaired Mann-Whitney U test	0.003519 to 0.05182	231	p=0.0245*

SR6a: Supplementary Figure 2: Vessel responses to neuronal calcium events from xy movies split by size (comparison of ROI type frequencies)

Figure Label	Test	Counts	X-squared	Degrees of Freedom	P value
**SD 2d (<=7µm)**	Pearson's Chi-squared test, 2x2 contingency table	HC: R-45, NR-254<br>V1: R-154, NR-550	5.725	1	p=0.0152*
**SD 2j (>7µm)**	Pearson's Chi-squared test, 2x2 contingency table	HC: R-75, NR-405<br>V1: R-159, NR-375	27.721	1	p<0.0001*

SR6b: Supplementary Figure 2: Vessel responses to local neuronal calcium events split by vessel size (comparison of response sizes)

All two group comparisons were first subject to an F test to compare variances, those with equal variances were subject to independent sample t-tests, and those which showed significant differences between groups Mann-Whitney U tests.

Figure Label	Mean	Standard Deviation	Test	Test Statistic	95% Confidence Interval	Degrees of Freedom	P value
**SD 2b (Vessel diameters, <=7μm)**	HC: 5.826<br>V1: 5.293	HC: 0.7857<br>V1: 1.074	Two-tailed unpaired t-test	t=1.758	-1.145 to 0.08102	37	p=0.09
**SD 2b (Vessel diameters, >7μm)**	HC: 11.70<br>V1: 11.21	HC: 3.704<br>V1: 3.712	Two-tailed Mann-Whitney U test	u=248	9.518 to 12.90	46	p=0.4706
**SD 2e (NVC_{index}, <=7 μm)**	HC: 0.1083<br>V1: 0.1738	HC: 0.1303<br>V1: 0.2461	Two-tailed unpaired Mann-Whitney U test	u=3077	-0.007 to 0.040	177	p=0.2552
**SD 2h (calcium peaks, <=7 μm)**	HC: 0.9967<br>V1: 0.8448	HC: 1.319<br>V1: 1.135	Two-tailed unpaired Mann-Whitney U test	u=2959	-0.242 to 0.031	177	p=0.1373
**SD 2i (diameter peaks, <=7 μm)**	HC: 0.0395<br>V1: 0.0402	HC: 0.0374<br>V1: 0.0306	Two-tailed unpaired Mann-Whitney U test	u=3216	-0.0043 to 0.010	177	p=0.4660
**SD 2k (NVC_{index}, >7 μm)**	HC: 0.0860<br>V1: 0.2292	HC: 0.1598<br>V1: 0.2064	Two-tailed unpaired Mann-Whitney U test	u=2917	0.062 to 0.147	232	p=3E-10*
**SD 2n (calcium peaks, >7 μm)**	HC: 0.7287<br>V1: 0.6002	HC: 0.8293<br>V1: 0.8705	Two-tailed unpaired Mann-Whitney U test	u=4681	-0.2044 to -0.02736	232	p=0.0078*
**SD 2o (diameter peaks, >7 μm)**	HC: 0.0295<br>V1: 0.0578	HC: 0.035<br>V1: 0.050	Two-tailed unpaired Mann-Whitney U test	u=2857	0.0145 to 0.0259	232	p=1.3E-10*

SR7a: Supplementary Figure 3: The contribution of cellular input to vascular responses (comparison of ROI type frequencies)

Figure Label	Test	Counts	X-squared	Degrees of Freedom	P value
**SD 3b (ROI type distribution)**	Pearson's Chi-squared test, 2x2 contingency table	HC >60% NP: 30<br>HC >60% Soma: 10<br>V1 >60% NP: 32<br>V1 >60% Soma: 9	0.105	df=3	p=0.7976

SR7b: Supplementary Figure 3: The contribution of cellular input to vascular responses (comparison of response frequencies)

Figure Label	Test	Responsive	Non-Responsive	X-squared	Degrees of Freedom	P value
**SD 3d (vessel responses)**	Pearson's Chi-squared test, with post-hoc pairwise table	HC >60% NP: 66<br>HC >60% Soma: 39<br>V1 >60% NP: 271<br>V1 >60% Soma: 42	HC >60% NP: 401<br>HC >60% Soma: 175<br>V1 >60% NP: 737 V1 >60% Soma: 188	34.707	df=3	p=1.4E-7*

Comparison	P value	Adjusted P Value
**HC >60% NP vs. HC >60% Soma**	p=0.172	p=0.217
**V1 >60% NP vs. V1 >60% Soma**	p=0.007*	p=0.0148*
**HC >60% Soma vs. V1 >60% Soma**	p=1.00	p=1.00
**HC >60% NP vs. V1 >60% NP**	p=2.69E-8*	p=1.61E-7*

SR7c: Supplementary Figure 3: The contribution of cellular input to vascular responses (comparison of response sizes)

All two group comparisons were first subject to an F test to compare variances, those with equal variances were subject to independent sample t-tests, and those which showed significant differences between groups Mann-Whitney U tests.

Figure Label	Mean	Standard Deviation	Test	95% Confidence Interval	Degrees of Freedom	P value
**SD 3c (diameter)**	HC >60% NP: 8.8, HC >60% Soma: 10.6, V1 >60% NP: 7.9, V1 >60% Soma: 10	HC >60% NP: 3.8, HC >60% Soma: 5.04, V1 >60% NP: 4, V1 >60% Soma: 3.9	Two-tailed unpaired Mann-Whitney U test	HC: -1.238 to 4.774<br>V1: -0.5319 to 6.073	HC: df=29<br>V1: df=39	HC: p=0.2723<br>V1: p=0.1203
**SD 3e (HC calcium)**	>60% NP: 0.69<br>>60% Soma: 0.95	>60% NP: 0.72<br>>60% Soma: 1.50	Two-tailed unpaired Mann-Whitney test	-0.2089 to 0.1152	df=97	p=0.6354
**SD 3f (HC diameter)**	>60% NP: 0.023<br>>60% Soma: 0.018	>60% NP: 0.016<br>>60% Soma: 0.014	Two-tailed unpaired Mann-Whitney test	-0.009751 to -0.001060	df=97	p=0. 0185*
**SD 3g (HC NVC_{index})**	>60% NP: 0.090<br>>60% Soma: 0.041	>60% NP: 0.179<br>>60% Soma: 0.031	Two-tailed unpaired Mann-Whitney test	-0.02401 to 0.003548	df=97	p=0.1688
**SD 3h (V1 calcium)**	>60% NP: 0.735<br>>60% Soma: 0.481	>60% NP: 1.066<br>>60% Soma: 0.512	Two-tailed unpaired Mann-Whitney test	-0.08000 to 0.07744	df=311	p=0.8330
**SD 3i (V1 diameter)**	>60% NP: 0.042<br>>60% Soma: 0.049	>60% NP: 0.039<br>>60% Soma: 0.060	Two-tailed unpaired Mann-Whitney test	-0.009962 to 0.005523	df=311	p=0.5657
**SD 3j (V1 NVC_{index})**	>60% NP: 0.186<br>>60% Soma: 0.158	>60% NP: 0.220<br>>60% Soma: 0.170	Two-tailed unpaired Mann-Whitney test	-0.03969 to 0.02685	df=311	p=0.8601

SR7d: Supplementary Figure 3: The contribution of cellular input to vascular responses (comparison of response sizes across region and ROI type)

We ran a multifactorial ANOVA on the calcium peaks, diameter peaks and NVC_{index} presented in supplementary figure 2 to look for effects of region and cellular input as well as any interactions, however the ANOVAs assumption of equal variances was violated, so we also ran multiple one-way ANOVAs with Welch correction (below).

Figure Label	Test	Test Statistic	Type III Sum of Squares	Degrees of Freedom	P value
**SD 3e, 3h (calcium)**	Multifactorial ANOVA: region, ROI type, region * ROI type interaction	Region: F=2.419, ROI Type: F=0.00002, Interaction: F=3.475	Region: 2.572, ROI Type: 2E-5, Interaction: 3.695	Region: df=1, ROI Type: df=1, Interaction: df=1	Region: p=0.121, ROI Type: p=0.997, Interaction: p=0.063 (trend)
**SD 3f, 3i (diameter)**	Multifactorial ANOVA: region, ROI type, region * ROI type interaction	Region: F=26.374, ROI Type: F=0.015, Interaction: F=1.676	Region: 0.037, ROI Type: 2.03E-5, Interaction: 0.002	Region: df=1, ROI Type: df=1, Interaction: df=1	Region: p=4.4E-7*, ROI Type: p=0.904, Interaction: p=0.196
**SD 3g, 3j (NVC_{index})**	Multifactorial ANOVA: region, ROI type, region * ROI type interaction	Region: F=16.374, ROI Type: F=2.113, Interaction: F=0.148	Region: 0.648, ROI Type: 0.084, Interaction: 0.006	Region: df=1, ROI Type: df=1, Interaction: df=1	Region: p=6E-4*, ROI Type: p=0.147 Interaction: p=0.700

The distribution of diameter peaks (p=4E-14) and NVC_{index} (p=8.9E-9) were unequal across brain regions, and the distribution of NVC_{index} values were also unequal across ROI types (p=0.001) (independent samples Mann-Whitney U test), meaning the variance differed across groups. Therefore, we also ran multiple one-way ANOVA tests using Welch's correction, which demonstrated significant effects of region on diameter and NVC_{index} (higher values in V1 vs HC), and significant effects of ROI type on NVC_{index} (higher values in NP vs soma).

Figure Label	Test	Test Statistic	Sum of Squares	Degrees of Freedom	P value
**SD 3f, 3i (diameter)**	Welch statistic	Region: F=26.513	Region: 0.574	410	Region: p=4.1E-7*
**SD 3g, 3j (NVC_{index})**	Welch statistic	Region: F=23.616, ROI Type: F=7.037	Region: 16.240, ROI Type: 16.886	410	Region: p=0.000002*, ROI Type: p=0.008*

SR8a: Supplementary Figure 4: The contribution of laminar organisation to vascular responses (comparison of response frequencies)

Figure Label	Test	Responsive	Non-Responsive	X-squared	Degrees of Freedom	P value
**SD 4a (HC vessel responses)**	Pearson's Chi-squared test, with post-hoc pairwise table	SO: 12<br>SP: 76<br>SR: 24<br>SLM: 8	SO: 80<br>SP: 391<br>SR: 133<br>SLM: 55	1.0202	df=3	p=0.7964

Comparison	P value	Adjusted P Value
**SO vs. SP**	p=0.535	p=1.000
**SO vs. SR**	p=0.765	p=1.000
**SO vs. SLM**	p=1.000	p=1.000
**SP vs. SR**	p=0.868	p=1.000
**SP vs. SLM**	p=0.585	p=1.000
**SR vs. SLM**	p=0.779	p=1.000

SR8b: Supplementary Figure 4: The contribution of laminar organisation to vascular responses (comparison of frequency of observations in each layer)

Figure Label	Test	Responsive	Non-Responsive	X-squared	Degrees of Freedom	P value
**SD 4c (V1 vessel responses)**	Pearson's Chi-squared test, 2x2 contingency table	L1: 48<br>L2/3: 261	L1: 164<br>L2/3: 747	0.81469	df=1	p=0.3667

SR8c: Supplementary Figure 4: The contribution of laminar organisation to vascular responses (comparison of vessel sizes across HC layers)

Figure Label	Mean	Standard Deviation	Test	Test Statistic	Degrees of Freedom	P value
**SD 4b (HC vessel sizes)**	SO: 9.6914<br>SP: 8.6245<br>SR: 8.1862<br>SLM: 9.117	SO: 3.4501<br>SP: 3.2296<br>SR: 3.7051<br>SLM: 5.4891	One-way ANOVA with Bonferroni post-hoc comparisons	F=0.2487	df=3	p=0.8517

Comparison	95% Confidence Interval	Adjusted P Value
**SO vs. SP**	-3.276 to 5.410	p>0.999
**SO vs. SR**	-3.675 to 6.685	p>0.999
**SO vs. SLM**	-5.699 to 6.847	p>0.999
**SP vs. SR**	-3.694 to 4.570	p>0.999
**SP vs. SLM**	-5.933 to 4.947	p>0.999
**SR vs. SLM**	-7.060 to 5.198	p>0.999

When comparing layers in HC, we tested the assumption of equal variances (independent samples Kurskal-Wallis test). The distribution of calcium peaks (p=0.057) and NVC_{index} (p=0.494) were equal between layers, but diameter peak (p=0.002) distribution was different across layers. For calcium peaks and NVCindex we ran one-way ANOVAs with Bonferroni post-hoc comparisons, however as diameter peak data violated the assumptions of a normal one-way ANOVA, We ran a one-way ANOVA with an adjusted F test (Welch statistic), with Games-Howell post-hoc comparisons (i.e. where unlike Bonferroni post-hoc tests, equal variances are not assumed).

SR8d: Supplementary Figure 4: The contribution of laminar organisation to vascular responses (comparison of calcium response sizes in HC)

Figure Label	Mean	Standard Deviation	Test	Test Statistic	Degrees of Freedom	P value
**SD 4e (HC calcium)**	SO: 1.1780<br>SP: 0.7840<br>SR: 0.6604<br>SLM: 0.5236	SO: 1.0884<br>SP: 1.1693<br>SR: 0.5042<br>SLM: 0.4752	One-way ANOVA with Bonferroni post-hoc comparisons	F=0.8710	df=3	p=0.5154

Comparison	95% Confidence Interval	Adjusted P Value
**SO vs. SP**	-0.4647 to 1.253	p>0.9999
**SO vs. SR**	-0.4597 to 1.495	p=0.9471
**SO vs. SLM**	-0.6072 to 1.916	p=0.9990
**SP vs. SR**	-0.5236 to 0.7708	p>0.9999
**SP vs. SLM**	-0.7669 to 1.288	p>0.9999
**SR vs. SLM**	-0.9916 to 1.265	p>0.9999

SR8e: Supplementary Figure 4: The contribution of laminar organisation to vascular responses (comparison of vascular response sizes in HC)

Figure Label	Mean	Standard Deviation	Test	Test Statistic	Degrees of Freedom	P value
**SD 3f (HC diameter)**	SO: 0.0586<br>SP: 0.0278<br>SR: 0.0503<br>SLM: 0.0454	SO: 0.0693<br>SP: 0.0208<br>SR: 0.0445<br>SLM: 0.0363	One-way ANOVA with Welch statistic with, and Games-Howell post-hoc comparisons	F=4.630	df=3	p=0.004*

Comparison	95% Confidence Interval	Adjusted P Value
**SO vs. SP**	-0.0296 to 0.0911	p=0.454
**SO vs. SR**	-0.0547 to 0.713	p=0.981
**SO vs. SLM**	-0.0545 to 0.0809	p=0.944
**SP vs. SR**	-0.0482 to 0.0033	p=0.103
**SP vs. SLM**	-0.0606 to 0.0255	p=0.574
**SR vs. SLM**	-0.0508 to 0.0411	p=0.989

Whilst we observed a general effect of layer in HC (one way ANOVA with Welch's statistic), this effect was lost after multiple post-hoc comparisons (Games-Howell), and could not be attributed to any specific layer to layer combination. This could be due to a lack of statistical power (from small group sizes) and/or a high number of factor levels (meaning we are more likely to accept the null hypothesis).
Link to a relevant discussion thread:
https://www.researchgate.net/post/Is_it_possible_to_get_non_significant_results_in_post_hoc_test_when_we_got_the_significant_result_in_ANOVA

SR8g: Supplementary Figure 4: The contribution of laminar organisation to vascular responses (comparison of NVC_{index} in HC)

Figure Label	Mean	Standard Deviation	Test	Test Statistic	Degrees of Freedom	P value
**SD 4g (HC NVC_{index})**	SO: 0.0775<br>SP: 0.1149<br>SR: 0.1251<br>SLM: 0.1182	SO: 0.0759<br>SP: 0.1827<br>SR: 0.1302<br>SLM: 0.1318	One-way ANOVA with Bonferroni post-hoc comparisons	F=0.2403	df=3	p=0.8681

Comparison	95% Confidence Interval	Adjusted P Value
**SO vs. SP**	-0.1733 to 0.09839	p>0.9999
**SO vs. SR**	-0.2022 to 0.1070	p>0.9999
**SO vs. SLM**	-0.2403 to 0.1589	p>0.9999
**SP vs. SR**	-0.1126 to 0.09219	p>0.9999
**SP vs. SLM**	-0.1658 to 0.1592	p>0.9999
**SR vs. SLM**	-0.1716 to 0.1854	p>0.9999

SR8h: Supplementary Figure 4: The contribution of laminar organisation to vascular responses (comparison of response sizes in V1)

All two group comparisons were first subject to an F test to compare variances, those with equal variances were subject to independent sample t-tests, and those which showed significant differences between groups Mann-Whitney U tests.

Figure Label	Mean	Standard Deviation	Test	Test Statistic	95% Confidence Interval	Degrees of Freedom	P value
**SD 4d (V1 vessel sizes)**	L1: 9.7921<br>L2/3: 7.9863	L1: 4.8976<br>L2/3: 3.8780	Two-tailed unpaired t-test	t=1.118	-5.075 to 1.463	df=38	p=0.2704
**SD 4h (V1 calcium)**	L1: 0.6273<br>L2/3: 0.6876	L1: 1.0405<br>L2/3: 0.9847	Two-tailed unpaired Mann-Whitney test	NA	-0.007055 to 0.1327	df=307	p=0.0777 (trend)
**SD 4i (V1 diameter)**	L1: 0.0778<br>L2/3: 0.0477	L1: 0.0524<br>L2/3: 0.0396	Two-tailed unpaired Mann-Whitney test	NA	-0.03944 to -0.01622	df=307	p=0.0000045*
**SD 4j (V1 NVC_{index})**	L1: 0.4181<br>L2/3: 0.1907	L1: 0.3335<br>L2/3: 0.2230	Two-tailed unpaired Mann-Whitney test	NA	-0.2890 to -0.09624	df=307	p=0.000011*

SR8i: Supplementary Figure 4: The contribution of laminar organisation to vascular responses (comparison of response sizes between regions)

When comparing regions, we could not compare all the separate layers of HC (i.e. SO, SP, SR, SLM) to those of V1 (i.e. L1, L2/3), as they are organised differently in each region. Because there were no differences between layers in HC, we therefore collapsed across layers in in this region, and compared to each of the layers of V1. We first tested the assumption of equal variances between layers (i.e. HC all layers, V1 L1, V1 L2/3) (independent samples Kruskal-Wallis test). The distribution of calcium peaks (p=0.005), diameter peaks (p<0.001) and NVC_{index} (p<0.001) was different across these layers. We therefore ran one-way ANOVAs with an adjusted F test (Welch statistic), with Games-Howell post-hoc comparisons (i.e. where unlike Bonferroni post-hoc tests, equal variances are not assumed).

Descriptives:

Dependent Variable	Mean	Standard Deviation
**Calcium Peaks**	HC: 0.7813, V1 L1: 0.6273, V1 L2/3: 0.6876	HC: 1.03, V1 L1: 1.04, V1 L2/3: 0.985
**Diameter Peaks**	HC: 0.0366, V1 L1: 0.0778, V1 L2/3: 0.0477	HC: 0.036, V1 L1: 0.052, V1 L2/3: 0.040
**NVC_{index}**	HC: 0.113, V1 L1: 0.418, V1 L2/3: 0.191	HC: 0.161, V1 L1: 0.333, V1 L2/3: 0.223

Figure Label	Test	Test Statistic	Sum of Squares	Degrees of Freedom	P value
**SD 4e-j**	One-way ANOVA with Welch statistic, and Games-Howell post-hoc comparisons	Calcium: F=0.53, Diameter: F=17.939, NVC_{index}: F=32.011	Calcium: 1.066, Diameter: 0.058, NVC_{index}: 3.193	2	Calcium: p=0.589, Diameter: p=3.3E-8*, NVC_{index}: 1.1E-13*

Comparison	95% Confidence Interval	P Value
**Calcium:**		
HC vs. L1	-0.2683 to 0.5764	p=0.661
HC vs. L2/3	-0.1703 to 0.3578	p=0.680
L1 vs. L2/3	-0.4492 to 0.3287	p=0.927
**Diameter:**		
HC vs. L1	-0.0610 to -0.0214	p=0.000014*
HC vs. L2/3	-0.0208 to -0.0014	p=0.02*
L1 vs. L2/3	0.0110 to 0.0492	p=0.001*
**NVC_{index}:**		
HC vs. L1	-0.4259 to -0.1835	p=3.7E-7*
HC vs. L2/3	-0.1248 to -0.0298	p=0.00045*
L1 vs. L2/3	0.1068 to 0.3480	p=0.00009*

SR9: Supplementary Figure 5: The contribution of hippocampal cranial window surgery

The paired t test analyses the differences between pairs, in cases were the difference between pairs was not a consistent measure of effect, the ratio paired t-test was a more consistent way to quantify the effect. All paired comparisons were also subject to a distribution test, and those with unequal variances were compared using a Wilcoxon matched-pairs signed rank test.

Figure Label	Test	95% Confidence Interval	Degrees of Freedom	P value
**SD 5b (CA1 area)**	Ratio paired t-test	0.8565 to 1.346	11	p=0.4558
**SD 5c (aspect ratio)**	Ratio paired t-test	0.5788 to 1.068	11	p=0.0993
**SD 5d (vascular density)**	Wilcoxon matched-pairs test	-0.2571 to 0.5366	11	p=0.4375

SR10a: Supplementary Figure 6: The impact of hippocampal cranial window surgery (comparison of response sizes)

All two group comparisons were first subject to an F test to compare variances, those with equal variances were subject to independent sample t-tests, and those which showed significant differences between groups Mann-Whitney U tests.

Figure Label	Mean	Standard Deviation	Test	Test Statistic	95% Confidence Interval	Degrees of Freedom	P value
**SD 6d (behaviour)**	HC: 65.8<br>V1: 67	HC: 11.09<br>V1: 15.41	Two-tailed unpaired t-test	t=-0.214	-13.88 to 11.48	9	p=0.8352
**SD 6f (vessel sizes)**	HC <100µm: 9.23, HC >110µm: 9.72<br>V1 <100µm: 9.08, V1 >110µm: 7.79	HC <100µm: 3.87, HC >110µm: 4.61<br>V1 <100µm: 4.77, V1 >110µm: 3.46	Two-tailed unpaired Mann-Whitney test	HC: u=208<br>V1: u=177	HC: -2.084 to 2.220<br>V1: -3.136 to 1.463	HC: df=43<br>V1: df=39	HC: p=0.83<br>V1: p=0.49
**SD 6i (HC calcium)**	<100µm: 0.84<br>>110µm: 0.68	<100µm: 1.21<br>>110µm: 0.52	Two-tailed unpaired Mann-Whitney U test	u=1433	-0.06908 to 0.2156	118	p=0.3047
**SD 6j (HC diameter)**	<100µm: 0.03<br>>110µm: 0.04	<100µm: 0.03<br>>110µm: 0.04	Two-tailed unpaired t-test	u=1289	-0.0003854 to 0.01330	118	p=0.07 (trend)
**SD 6k (HC NVC_{index})**	<100µm: 0.10<br>>110µm: 0.13	<100µm: 0.12<br>>110µm: 0.22	Two-tailed unpaired Mann-Whitney U test	u=1507	-0.01347 to 0.02855	118	p=0.5370
**SD 6m (V1 calcium)**	<100µm: 0.73<br>>110µm: 0.69	<100µm: 1.02<br>>110µm: 1.01	Two-tailed unpaired Mann-Whitney U test	u=11108	-0.06064 to 0.05550	311	p=0.99
**SD 6n (V1 diameter)**	<100µm: 0.05<br>>110µm: 0.05	<100µm: 0.05<br>>110µm: 0.04	Two-tailed unpaired Mann-Whitney U test	u=11029	-0.007639 to 0.008108	311	p=0.9076
**SD 6o (V1 NVC_{index})**	<100µm: 0.24<br>>110µm: 0.22	<100µm: 0.28<br>>110µm: 0.24	Two-tailed unpaired Mann-Whitney U test	u=10559	-0.01720 to 0.03885	311	p=0.46

SR10b: Supplementary Figure 6: The impact of hippocampal cranial window surgery (comparison of vessel depth distribution)

Figure Label	Test	Counts	X-squared	Degrees of Freedom	P value
**SD 6e (depth distribution)**	Pearson's Chi-squared test	HC: <100µm 81, >110µm 36<br>V1: <100µm 130, >110µm 183	26.1423	3	p<0.00001*

SR10c: Supplementary Figure 6: The impact of hippocampal cranial window surgery (comparison of response frequencies)

Figure Label	Test	Responsive	Non-Responsive	X-squared	Degrees of Freedom	P value
**SD 6g (vessel responses)**	Pearson's Chi-squared test, with post-hoc pairwise table	HC <100µm: 79, HC >110µm: 35, V1 <100µm: 109, V1 >110µm: 204	HC <100µm: 448, HC >110µm: 186,<br>V1 <100µm: 363, V1 >110µm: 562	30.092	3	p=1.32E-6*

Comparison	P value	Adjusted P Value
**HC <100µm vs. HC >110µm**	p=0.855	p=0.855
**HC <100µm vs. V1 <100µm**	p=0.00142*	p=0.00284*
**HC >110µm vs. V1 >110µm**	p=0.00132*	p=0.00284*
**V1 <100µm vs. V1 >110µm**	p=0.185	p=0.222

SR11a: Supplementary Figure 7: The contribution of distance from perfusion source on vascular responses (comparison of distances and vascular responses)

All two group comparisons were first subject to an F test to compare variances, those with equal variances were subject to independent sample t-tests, and those which showed significant differences between groups Mann-Whitney U tests.
When comparing the vessel diameters sampled separated by region and vessel responsivity (i.e. responsive or not responsive to preceding calcium) (Supplementary Figure 5D), we found that the distribution of distance measurements between brain regions (p<0.001) was unequal (independent samples Mann-Whitney U test). Therefore, for Supplementary Figure 5D, we ran an alternative one-way ANOVA with a Welch correction.

Figure Label	Mean	Standard Deviation	Test	Test Statistic	95% Confidence Interval	Degrees of Freedom	P value
**SD 7b (RBCV, distance)**	HC: 226.5, V1: 134.6	HC: 73.4, V1: 86.76	Two-tailed unpaired Mann-Whitney test	NA	-133.0 to -59.4	115	p=7.5E-6*
**SD 7d (diameter, distance)**	HC: 220.4388, V1: 136.5902	HC: 63.12676, V1: 76.19212	One-way ANOVA with Welch statistic	F=658.819	165.3817 to 172.5662	2015	p=5.96E-126*
**SD 7g (V1, diameter)**	<=125µm: 0.05058, >=225µm: 0.05125	<=125µm: 0.04535, >=225µm: 0.05734	Two-tailed unpaired Mann-Whitney test	NA	-0.01688 to 0.008758	140	p=0.5098
**SD 7i (HC, diameter)**	<=125µm: 0.03935, >=225µm: 0.03101	<=125µm: 0.03154, >=225µm: 0.03541	Two-tailed unpaired Mann-Whitney test	NA	-0.02162 to 0.007383	77	p=0.4102

SR11b: Supplementary Figure 7: The contribution of distance from perfusion source on vascular responses (linear regression of haemodynamic measures sorted by distance to source)

Figure Label	Test	Test Statistic	95% Confidence Interval (Slope)	Equation	Degrees of Freedom	P value
**SD 7c (RBCV)**	Linear regression	HC: F=0.02215, V1: F=4.168	HC: -0.003638 to 0.003135, V1: -0.01492 to -0.0001214	HC: Y = -0.0002515*X + 1.145, V1: Y = -0.007521*X + 2.908	HC: 55, V1: 50	HC: p=0.8822, V1: p=0.0465*
**SD 7c (RBCV)**	ANCOVA	Slope: F=0.2475, Elevation: F=12.51			112	Slope: p=0.6198, Elevation: p=0.0006*
**SD 7e (diameter)**	Linear regression	HC: F=0.1989, V1: F=2.565	HC: -0.0001182 to 7.478e-005, V1: -0.0001178 to 1.208e-005	HC: Y = -2.173e-005*X + 0.03794, V1: Y = 15.285e-005*X + 0.05644	HC: 118, V1: 311	HC: p=0.6565, V1: p=0.1103
**SD 7e (diameter)**	ANCOVA	Slope: F=3.515, Elevation: F=4.168			105	Slope: p=0.0636 (trend), Elevation: p=0.0437*

Supplementary Figure 8: Neurovascular pathways in vascular cells

For Supplementary Figures 8a-i, see Supplementary Data Tables 4-5.

SR12a: Supplementary Figure 10: Testing the reliability of the Oxy-CBF probe (comparison of baseline haemodynamic measures between animals)

Figure Label	Test	Test Statistic	Mean Square	Degrees of Freedom	P value
**SD 10a – comparing means**	One way ANOVA (factor: animal ID)	CMRO2: HC F=1.84, V1 F=1.48<br>CBF: HC F=1.971, V1 F=0.71<br>SO2, HC F=0.744, V1 F=0.95<br>Hbr, HC F=0.771, V1 F=1.87<br>Hbt, HC F=0.891, V1 F=0.85	CMRO2: HC 8716.8, V1 4063.7<br>CBF: HC 19090.5, V1 19121.7<br>SO2: HC 19.4, V1 53.6<br>Hbr: HC 985.4, V1 114.7<br>Hbt: HC 1447.2 V1 362.7	HC 8<br>V1 8	CMRO2: HC p=0.16, V1 p=0.29<br>CBF: HC p=0.14, V1 p=0.68<br>SO2: HC p=0.65, V1 p=0.53<br>Hbr: HC p=0.64, V1 p=0.19<br>Hbt: HC p=0.55, V1 p=0.59
**SD 10a – comparing standard deviations**	Multiple t-tests with Bonferonni correction	CMRO2: t=0.094,<br>CBF: t=-0.90,<br>SO2: t=-0.91,<br>Hbt: t=0.61<br>Hbr: t=1.44		11	CMRO2: p=0.93, after Bonf corr. p=0.93,<br>CBF: p=0.39, after Bonf corr. p=1.16,<br>SO2: p=0.39, after Bonf corr. p=1.53.<br>Hbt: p=0.55, after Bonf corr. p=1.10,<br>Hbr: p=0.19, after Bonf corr. p=0.89

SR12b: Supplementary Figure 10: Testing the reliability of the Oxy-CBF probe (comparison of response sizes in fluorescent versus non-fluorescent, or pre- and post-pentobarbital injection)

Figure Label	Mean	Standard Deviation	Test	Test Statistic	95% Confidence Interval	Degrees of Freedom	P value
**SD 10b (resting $CMRO_2$, CBF and SO_2)**	$CMRO_2$ – WT: 190.6 FL: 185.7 CBF – WT: 442.5 FL: 404.2 SO_2 – WT: 56.08 FL: 53.84	$CMRO_2$ – WT: 50.58 FL: 45.25 CBF – WT: 131.1 FL: 97.39 SO_2 – WT: 3.78 FL: 5.372	Two-tailed unpaired t-test	$CMRO_2$: t=0.1738, CBF: t=0.5911, SO_2: t=0.7478	$CMRO_2$: -66.73 to 56.97, CBF: -180.6 to 104.1 SO_2: -8.842 to 4.357	df=11	$CMRO_2$: p=0.8652, CBF: p=0.5664, SO_2: p=0.4703
**SD 10c ($CMRO_2$, CBF and SO_2 peaks)**	CBF – WT: 10.72 FL: 12.63 SO_2 – WT: 6.66 FL: 6.39 Hbt – WT: 4.30 FL: 4.61	CBF – WT: 2.89 FL: 5.61 SO_2 – WT: 1.48 FL: 1.90 Hbt – WT: 1.45 FL: 1.90	Two-tailed unpaired t-test	CBF: t=0.5978 SO_2: t=0.1046 Hbt: t=0.2438	CBF: -6.318 to 10.15 SO_2: -6.773 to 6.244 Hbt: -2.923 to 3.536	df=5	CBF: p=0.5760 SO_2: p=0.9207 Hbt: P=0.8170
**SD 10d ($CMRO_2$, CBF and SO_2 pre- and post-pentobarbital injection)**	CMRO2 – Pre: 266.9 Post: 5.65 CBF – Pre: 540.5 Post: 5.65 SO_2 – Pre: 51.49 Post: 0	CMRO2 – Pre: 95.77 Post: 3.78 CBF – Pre: 149.4 Post: 3.78 SO_2 – Pre: 5.27 Post: 0	Two-tailed unpaired t-test	CMRO2: t=4.720 CBF: t=6.200 SO2: t=16.93	CMRO2: -414.9 to -107.6 CBF: -774.3 to -295.3 SO_2: -59.94 to -43.05	df=4	CMRO2: p=0.0092* CBF: p=0.0034* SO_2: p=7.1E-5*
